# Auxin‐Producing *Pseudomonas* Recruited by Root Flavonoids Increases Rice Rhizosheath Formation through the Bacterial Histidine Kinase Under Soil Drying

**DOI:** 10.1002/advs.202500607

**Published:** 2025-06-19

**Authors:** Feiyun Xu, Yongsen Wang, Jinyong Yang, Xue Zhang, Ke Wang, Fan Ding, Jiayin Pang, Lu Tong, Chuqi Bai, Shu Chen, Leyun Sun, Chongxuan Du, Ju Fang, Mengqiang Xu, Liang Li, Xin Yu, Jiahong Gengli, Jianping Liu, Qian Zhang, Zhengrui Wang, Yiyong Zhu, Huanyuan Zhang‐Zheng, Jianhua Zhang, Weifeng Xu

**Affiliations:** ^1^ Center for Plant Water‐use and Nutrition Regulation State Key Laboratory of Agricultural and Forestry Biosecurity College of JunCao Science and Ecology Fujian Agriculture and Forestry University Fuzhou 350002 China; ^2^ Department of Ecology School of Life Sciences Nanjing University Nanjing 210000 China; ^3^ Institute of Resources Environment and Soil Fertilizer Fujian Academy of Agricultural Sciences Fuzhou 350013 China; ^4^ College of Resources and Environment Fujian Agriculture and Forestry University Fuzhou 350002 China; ^5^ The UWA Institute of Agriculture The University of Western Australia Perth WA 6009 Australia; ^6^ Jiangsu Collaborative Innovation Center for Solid Organic Waste Resource Utilization College of Resources and Environment Sciences Nanjing Agricultural, University Nanjing 210095 China; ^7^ Environmental Change Institute School of Geography and the Environment University of Oxford Oxford OX12JD UK; ^8^ Leverhulme Centre for Nature Recovery University of Oxford Oxford OX12JD UK; ^9^ State Key Laboratory of Agrobiotechnology The Chinese University of Hong Kong Shenzhen 518172 China

**Keywords:** polyploidy, pseudomonas, rhizosheath formation, rice, soil drying

## Abstract

Rhizosheath formation is facilitated by root hair length, root exudates, the soil microbes, which collectively enhance plant resistance to drought. This process partly results from the complex interaction between root exudates and microbes, a relationship that remains poorly understood. The roles of root exudates and microbes in rhizosheath formation in rice under soil drying (SD) conditions are investigated. In tetraploid rice, rhizosheath formation under SD is approximately 70% greater than in diploid rice. Inoculation of diploid rice with the rhizosheath soil microbiota from tetraploid rice significantly enhanced rhizosheath formation under SD. The bacterial genus *Pseudomonas* is identified as the key taxon promoting rhizosheath formation in tetraploid rice under SD. Tetraploid rice exhibits significantly higher root flavonoid concentration than diploid rice under SD. Overexpression of the chalcone synthase gene (*OsCHS1*), a key gene involved in flavonoid biosynthesis, led to a significant increase in the abundance of Pseudomonadaceae in diploid rice. *Pseudomonas nitroreducens*, isolated from the rhizosheath of tetraploid rice, demonstrates chemotactic attraction to flavonoids, but this behavior is not observed in histidine kinase mutant Δ*cheA*. Diploid and tetraploid rice inoculated with *P. nitroreducens* and IAA biosynthesis complemented strain Δ*iaaM*‐c formed larger rhizosheath under SD than those inoculated with its IAA biosynthesis mutant Δ*iaaM*. These results suggest that auxin‐producing *Pseudomonas*, recruited by root flavonoids, enhances rice rhizosheath formation through the bacterial histidine kinase under SD. This finding may facilitate the improvement of environmental adaptation in polyploidy crops by regulating their interactions with beneficial soil microorganisms.

## Graphical abstract

Drought is a major cause of crop losses across agricultural regions. Rhizosheath formation enhances plant resistance to drought. Polyploids may tolerate stress more effectively through increased interactions with bacteria. However, the contributions of tetraploid rice associated microbiota to rhizosheath formation under soil drying remain unclear. Auxin‐producing *Pseudomonas*, recruited by root flavonoids, enhances rice rhizosheath formation through the bacterial histidine kinase under soil drying.

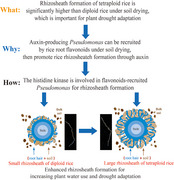

## 1 Introduction

Drought is a major cause of crop losses across agricultural regions.^[^ ^1^ ^]^ Climate change has increased the frequency of extreme weather events, such as reduced rainfall.^[^ ^2^, ^3^ ^]^ By 2050, approximately 50% of global regions are projected to experience water scarcity.^[^ ^4^ ^]^ Therefore, the development of crops with high yields and improved water‐use efficiency is critical for ensuring food security under changing climate conditions.^[^ ^4^ ^]^

Rhizosheath, an adaptive trait observed in desert species, is the soil that adheres to roots.^[^ ^5^, ^6^, ^7^ ^]^ It is defined as the weight of soil remaining attached to roots upon excavation.^[^ ^8^ ^]^ The rhizosheath retains a higher water content than bulk soil, thereby contributing to water uptake.^[^ ^9^ ^]^ Within the rhizosheath, mucilage can moderate the matric potential gradient between the rhizosheath‐soil interface and the bulk soil, allowing plants to maintain a continuous pathway for water acquisition.^[^ ^10^ ^]^ Mucilage also reduces the air gaps around roots, enhancing their water uptake capacity.^[^ ^11^ ^]^ Soil adherence to roots contributes to aggregate stability, which is positively associated with water retention.^[^ ^12^ ^]^ Wheat cultivars with large rhizosheath exhibit higher transpiration rates under drought stress.^[^ ^13^ ^]^

Rhizosheath formation is associated with root‐hair cylinder volume in rice, barley and wheat.^[^ ^6^, ^14^, ^15^ ^]^ Initially documented in drought‐tolerant plants exposed to drought conditions, the rhizosheath serves as a dynamic zone for water and nutrient exchange.^[^ ^14^, ^16^, ^17^ ^]^ Roots encased in a thick rhizosheath can endure severe drought by remaining hydrated and dormant.^[^ ^18^ ^]^ Using non‐destructive three‐dimensional imaging and mathematical modeling, Schmidt et al. (2012)^[^ ^19^ ^]^ demonstrated that root‐soil contact is influenced by soil porosity and the size of aggregates surrounding roots. We previously reported that the porosity of rhizosheath soil in rice is significantly higher than that of bulk soil,^[^ ^20^ ^]^ suggesting that the rhizosheath facilitates water uptake by enhancing soil porosity. Rhizosheath formation is linked to drought resistance in rice^[^ ^7^, ^20^, ^21^, ^22^ ^]^ and represents a strategy to improve plant water‐use efficiency under drought conditions.^[^ ^16^ ^]^

Multiple factors influence rhizosheath formation, including root hair length and density, soil structure and mucilage.^[^ ^14^, ^23^ ^]^ For example, root hair mutant in barley and rice show minimal or no rhizosheath formation.^[^ ^8^, ^20^ ^]^ Moderate soil drying enhances rhizosheath formation by promoting root hair growth in barley and rice.^[^ ^20^, ^21^ ^]^ Furthermore, the interaction between the endophytic fungus *Piriformospora indica* and the bacterium *Bacillus cereus* has been shown to enhance rhizosheath formation in rice through auxin‐modulated root hair growth under soil drying (SD) conditions.^[^ ^7^ ^]^

Polyploidy plays a central role in ecological and evolutionary processes among flowering plants.^[^ ^24^, ^25^, ^26^ ^]^ Polyploid plants exhibit enhanced flexibility in response to environmental changes.^[^ ^25^, ^27^, ^28^ ^]^ For instance, tetraploid rice and citrange increase achieve greater tolerance to drought stress by modulating phytohormone response pathways.^[^ ^29^, ^30^ ^]^ In tetraploid *Arabidopsis*, alterations in cell proliferation and organ size enhance drought tolerance and reduce the transpiration rate.^[^ ^31^ ^]^ The expression of genes involved in redox homeostasis and stress responses is significantly up‐regulated in polyploid *Arabidopsis* under drought conditions.^[^ ^31^ ^]^ Compared with its tetraploid progenitor, hexaploid wheat displays greater salt tolerance due to enhanced sodium ion absorption.^[^ ^32^ ^]^ Tetraploid rice shows increased H^+^ efflux, contributing to its salt tolerance.^[^ ^33^ ^]^ Furthermore, DNA hypomethylation in tetraploid rice under salt stress induces the expression of stress‐responsive genes.^[^ ^34^ ^]^ Polyploids may tolerate stress more effectively through increased interactions with bacteria and fungi.^[^ ^35^ ^]^ Enhanced colonization by mycorrhizae in tetraploid poker alumroot promotes nutrient exchange relative to diploid counterparts.^[^ ^36^ ^]^ Polyploidy also enhances nitrogen fixation by improving legume rhizobium interactions.^[^ ^37^ ^]^ However, the contributions of tetraploid rice associated microbiota to rhizosheath formation under soil drying conditions remain unclear.

In this study, we examined rhizosheath formation in both diploid and tetraploid rice under SD conditions. The bacterial communities present in the phyllosphere, rhizosphere (under well‐watered, WW conditions), rhizosheath (under SD conditions), and root endosphere of diploid and tetraploid rice were explored to determine the role of microbes in rhizosheath formation during SD. Several bacterial strains were isolated their involvement roles in rhizosheath formation was evaluated using an IAA biosynthesis mutant and complementary strains. To investigate how tetraploid rice recruits *Pseudomonas*, we utilized RNA sequencing, metabonomic analysis, and a chemotaxis mutant/complemented strains. The results provide evidence that plant polyploidy can facilitate the recruitment of native soil bacteria through root flavonoid biosynthesis, thereby enhancing rice rhizosheath formation and water‐use efficiency under soil drying conditions. These findings will contribute to strategies for improving plant adaptation climate variability.

## 2 Experimental Section

### 2.1 Plant Materials and Growth Conditions

The tetraploid rice line Nipponbare‐4 ×, developed through colchicines treatment (0.05%) of the Nipponbare‐2 × background to induce chromosome doubling to form tetraploid rice, was used in this previous study.^[^ ^33^ ^]^ Tetraploid rice (4n = 48) was artificially synthesized from *O. sativa* L. (2n = 24). Rice seeds were surface‐sterilized with 1.5% (v/v) NaClO for 20 min, and then washed five times with double‐distilled water. Seeds were germinated in 1/2 MS nutrient medium for 3 d. Seedlings were transplanted into pots measuring 12‐cm in diameter and 14‐cm in height. The experiments were conducted in a greenhouse under a 14 h light (26 °C)/10 h dark (22 °C) photoperiod, with 60% (w/w) relative humidity and a photosynthetic photon flux density of 300 µmol photons m^−2^ s^−1^. The greenhouse was equipped with an efficient ventilation system.

### 2.2 Experiment Design

Soil was collected from a rice experimental field at Fujian Agriculture and Forestry University, Fuzhou, China (119°14′E, 26°5′N). The chemical characteristics of the soil are provided in Table S1 (Supporting Information). Air‐dried soil was sieved through a 4 mm mesh to remove coarse material and vegetative debris. Water treatments were conducted following established protocols.^[^ ^7^, ^20^, ^21^ ^]^ Half of the pots were maintained under well‐watered (WW) conditions with a 1–2 cm layer of water above the soil surface. The remaining pots were subjected to a SD conditions, where plants were irrigated every 3 d to 80% (w/w) of field capacity (FC) (Figure S1A, Supporting Information). The SD treatment reduced rice shoot dry weight and promoted root growth,^[^ ^7^ ^]^ indicating that rice plants were under drought stress when water was maintained at 80% FC. WW, SD and SD with the auxin efflux inhibitor (NPA) treatments were maintained for 3 weeks. After treatment, newly and fully expanded leaves were harvested for analysis of relative water content, as described by Heckwolf et al. (2011).^[^ ^38^ ^]^ Water‐use efficiency (WUE) was calculated as the ratio of shoot dry weight to the irrigation volume.^[^ ^39^ ^]^ Net photosynthetic rate and stomatal conductance were measured on the youngest fully expanded leaves were determined using an LI‐6400 portable photosynthesis system (LI‐COR Biosciences), as described by Heckwolf et al. (2011).^[^ ^38^ ^]^ Leaf intrinsic water‐use efficiency (iWUE) was calculated as the ratio of the net photosynthetic rate to stomatal conductance.^[^ ^40^ ^]^ Three replicates of the rice plants were selected for each measurement, and the experiments were repeated twice. For PEG6000 treatment, 15% PEG6000 was added in nutrient solution to assess the phenotype of rice seedlings to drought stress.

### 2.3 Measurements of Root Morphological Traits and Rhizosheath Soil Weight

Rice plants were carefully removed from pots and fixed on an incubator shaker (Crystal Technology & Industries, Inc., USA). They were shaken at 150 rpm min^−1^ for 1 min to remove loosely the non‐adhering soil. Soil that tightly adhered to roots upon excavation was defined as rhizosheath soil.^[^ ^7^ ^]^ Rhizosheath soil was collected by gently washing the roots with water in plastic dishes. The mixture of soil and water was dried at 105 °C for 3 d to determine the rhizosheath soil dry weight. The root system was scanned using an Epson Expression 1640 XL flatbed scanner (Epson UK, London, UK), and total root length was quantified using WinRHIZO software (Regent Instruments, Quebec City, QC, Canada). Specific rhizosheath soil dry weight was calculated as the dry weight of rhizosheath soil per plant divided by the total root length.^[^ ^7^, ^20^, ^41^ ^]^ An SMZ18 stereomicroscope with a DS‐U3 camera (Nikon) was used to capture images of root hairs. The lengths of 10 fully elongated root hairs per sample were measured using Image J software (National Institutes of Health, Bethesda, MD, USA; v1.8.0). The mean value of these 10 measurements was used as the representative root hair length for each root sample. The cross‐section of rhizosheath was taken using a sharp razor blade (Figure S1B, Supporting Information). Rhizosheath soil water content was calculated using the following formula:

Rhizosheath soil water content = (fresh weight of roots with attached rhizosheath soil − root fresh weight − rhizosheath dry weight)/rhizosheath dry weight.^[^ ^21^ ^]^

### 2.4 Measurement of Root Free Proline and Malondialdehyde (MDA)

Root free proline content was determined using the acid ninhydrin method with a standard curve, as described by Bates et al., (1973).^[^ ^42^ ^]^ Root MDA content was assessed using the protocol developed by Hodges et al., (1999).^[^ ^43^ ^]^ Fresh root tissue (0.5 g) was homogenized with 5 mL of 5% 2,4,6‐trichloroanisole, and centrifuged at 3000 rpm for 10 min. Subsequently, 2 mL of 0.67% 2,4,6‐trichloroanisole were added to the supernatant. The mixture was boiled for 30 min and centrifuged again. Absorbance values of the resulting supernatant were recorded at 532, 600 and 450 nm to calculate MDA content.

### 2.5 Collection and Inoculation of Rhizosheath Soil Suspension

The experiment was conducted according to methods described by Lu et al. (2018) ^[^ ^44^ ^]^ and Yu et al. (2021).^[^ ^45^ ^]^ Briefly, roots were rinsed with 50 mL of autoclaved double‐distilled water for 10 min to obtain the rhizosheath soil suspension. A 20 mL aliquot of this suspension was used to inoculate other rice plants. The SD treatment was applied as previously described. Three replicates of the rice plants were selected for each experimental measurement, and the experiments were repeated twice.

### 2.6 Plant and Soil Sample Collection and DNA Extraction for Bacterial Community Analysis

Soil samples were collected as previously described.^[^ ^7^ ^]^ Under SD conditions, roots at the seedling stage were carefully removed from pots, and rhizosheath soil was obtained. Under WW conditions, soil was collected from the same radius surrounding roots as the rhizosheath under SD; this was regarded as rhizosheath‐like soil, because rice does not form a rhizosheath under WW. Roots were washed in phosphate‐buffered saline (PBS‐S) buffer (130 mM NaCl, 7 mM Na_2_HPO_4_, 3 mM NaH_2_PO_4_, pH 7.0, and 0.02% (v/v) Silwet L‐7) in 50‐mL tubes.^[^ ^46^ ^]^ The buffer was centrifuged at 1,500 g for 20 min to collect the rhizosheath soil. Bulk soil was collected from unplanted control pots.

Root samples were surface‐sterilized with 1.5% (v/v) NaClO for 15 min after removal from PBS‐S, and then washed three times with sterilized water. Water used in the final rinse was plated on Luria‐Bertani medium to confirm the effectiveness of sterilization.^[^ ^47^ ^]^ The youngest fully expanded leaves were also collected for analysis.^[^ ^48^ ^]^ Soil, root and leaf samples intended for sequencing were stored at °80 °C. Total genomic DNA of rhizosheath soil, rhizosphere soil, bulk soil, root, and leaf was extracted from 0.5 g samples using the Mag‐Bind Soil DNA Kit (Omega Bio‐Tek). Gel electrophoresis and a NanoDrop One spectrophotometry (Thermo Scientific, Waltham, MA, USA) were used to determine DNA quality and quantity. Three replicates for each treatment were subjected to high‐throughput 16S rRNA gene sequencing.

### 2.7 Bacterial Community Analysis

The V5‐V7 region of the bacterial 16S rRNA gene was amplified using the 799F/1193R primers.^[^ ^7^ ^]^ Reactions were conducted in triplicate 50‐µL mixtures containing 25 µL of Phusion High‐Fidelity PCR Master Mix with HF buffer (New England Biolabs), 3 µL of primers (10 µM), 10 µL of template DNA, 6 µL of double‐distilled water, and 3 µL of dimethyl sulfoxide. PCR conditions were as follows: initial denaturation at 98 °C for 30 s; 25 cycles of 98 °C for 15 s, 58 °C for 15 s; and a final extension at 72 °C for 15 s, and 72 °C for 60 s. PCR products were extracted from 2% agarose gels, purified using the AxyPrep DNA Gel Extraction Kit (Axygen Biosciences, Union City, CA, USA), and quantified using Quanti‐Fluor‐ST (Promega, Madison, WI, USA). Sequencing was carried out on the MiSeq platform (Illumina, San Diego, CA, USA).

The DADA2 pipeline in QIIME2 was used to process raw 16S rRNA gene reads, including quality filtering, dereplication, denoising, merging and chimera removal.^[^ ^49^ ^]^ The resulting feature table was filtered to exclude amplicon sequence variants (ASVs) with frequencies <2.^[^ ^50^ ^]^ For leaf and root samples, ASVs assigned to mitochondria and chloroplasts were removed. Taxonomic classification of ASVs was performed using the QIIME2 naive Bayes classifier trained on 99% operational taxonomic units from the SILVA database (v138).^[^ ^51^ ^]^ Alpha‐diversity metrics (Chao1 and Shannon indices) and beta‐diversity metrics were calculated using Mothur^[^ ^52^ ^]^ and the R package vegan (v4.0.4). Rarefaction curves based on operational taxonomic units were generated to evaluate sampling efficiency. Linear discriminant analysis coupled with effect size (LEfSe) was used to identify taxa exhibiting significant difference in abundance (*p* <0.05) among treatments. All sequencing data were available in the NCBI BioProject database under accession number PRJNA859640 (Table S2, Supporting Information).

### 2.8 Bacterial Culture and Isolation

The population density of culturable *Pseudomonas* from fresh bulk soil, rhizosphere (under WW) and rhizosheath (under SD) was measured using a standard 10‐fold dilution plating method as described by Wei et al. (2019).^[^ ^53^ ^]^ Three aliquots of each dilution were plated onto cephaloridine‐fucidin‐cetrimide (CFC) *Pseudomonas* semi‐selective medium.^[^ ^54^ ^]^ Plates were incubated at 30 °C for 2 d, and colony forming units (CFUs) were counted to determine population densities.

To isolate putative *Pseudomonas* strains, fresh rhizosheath soil was collected form tetraploid rice under SD. The soil suspension was serially diluted and plated onto CFC *Pseudomonas* semi‐selective medium.^[^ ^55^ ^]^ After incubation at 30 °C for 2 d, colonies were selected at random based on morphological characteristics. A total of 54 candidate *Pseudomonas* strains were isolated from the rhizosheath soil. Interactive isolates were identified by 16S rRNA gene sequencing using primers 27F and 1492R; sequencing was performed at Sangon Biotech (Shanghai) Co., Ltd.

### 2.9 Root RNA‐Seq Analysis

Total RNA was extracted using TRIzol reagent (Invitrogen, Carlsbad, CA, USA) following the protocol of Xu et al. (2019).^[^ ^56^ ^]^ RNA quality was assessed using the Agilent 2100 Bioanalyser and concentration was determined using the NanoDrop spectrophotometer (Thermo Fisher Scientific). The RNA‐seq library was prepared using the TruSeq RNA Sample Preparation Kit (Illumina, San Diego, CA, USA) and sequenced on the Illumina NovaSeq 6000 platform at Major Bio‐pharm Technology Co., Ltd, (Shanghai, China). Raw sequencing reads were processed and cleaned using SOAPnuke (v1.5.2). High‐quality reads were mapped to the *Oryza_sativa*_Japonica_Group reference genome (IRGSP‐1.0). Transcript abundance was quantified using Bowtie2 (v2.4.1) and expressed in fragments per kilobase of transcript per million mapped fragments reads (FPKM). Differentially expressed genes (DEGs) were identified based on false discovery rate <0.05 and fold change ≥ 2. Pathway enrichment analysis of DEGs was performed using the Kyoto Encyclopedia of Genes and Genomes (KEGG) database. Three replicates of each sample were used for the transcriptome analysis. Sequencing data have been deposited in the NCBI BioProject database under accession number PRJNA859542 (Table S2, Supporting Information).

### 2.10 Analysis of Flavonoid and Auxin Concentrations

Flavonoid concentration in rice roots at the seedling stage were analyzed according to the method of Dai et al. (2019).^[^ ^57^ ^]^ Briefly, 1 g root was extracted in 10 mL of 50% ethanol for 1 h and brought to a final volume of 50 mL. The supernatant was followed by the addition of NaNO_2_ and AlCl_3_. 1 M NaOH were added, and the final volume was adjusted to 10 mL with double‐distilled water. Finally, absorbance was measured at 510 nm, and the solvent mixture served as a blank control. To quantify auxin content in *Pseudomonas* isolates, the culture supernatant was filtered through standard filter paper. The resulting filtrate was used to determine auxin concentrations via HPLC following the protocol of Yuan et al. (2013).^[^ ^58^ ^]^ Three replicates were selected for each experimental measurement, and experiments were repeated twice.

### 2.11 Metabolomics Analysis

For untargeted metabolomics analysis, root tissues grown under SD were frozen in liquid nitrogen. The homogenized tissue was resuspended in 70% methanol aqueous extract (with an internal standard). For flavonoid‐specific metabolomics, roots were pooled and rinsed thoroughly three times with sterilized water. Roots were then transferred to a cylinder containing sterilized distilled water for the collection of root exudates. After 24 h, the root exudates were filtered through a 0.2‐µm membrane. Metabolite analysis was performed using a UPLC‐ESI‐MS/MS system (UPLC, Shim‐pack UFLC SHIMADZU CBM30A; MS, Applied Biosystems 4500 Q TRAP) at Wuhan MetWare Biotechnology Co., Ltd. (China).^[^ ^59^, ^60^, ^61^ ^]^ Three biological replicates of each treatment were taken for the analysis of metabolites.

### 2.12 Effect of Flavonoids on Pseudomonas Growth and Rhizosheath Formation

To investigate the effects of flavonoids on *Pseudomonas* growth, rhizosheath formation and WUE, flavonoids were applied at a concentration of 1 µM. Sterilized rice seedlings were transplanted into pots filled with either natural (non‐sterilized) soil or sterilized soil. The sterilized soil was prepared through triple autoclaving and heat‐incubation until completely dehydrated.^[^ ^62^ ^]^ Twenty mL of flavonoid solution were added to each pot once per week for 3 weeks. Total root length, root hair length and rhizosheath soil weight were measured as described earlier. Three replicates of the rice plants were selected for each experimental measurement, and experiments were repeated twice.

### 2.13 Genome Sequencing of PSE14 and Mutants, Construction of Complementary Strains

Genomic DNA from the PSE14 was extracted using the SDS method. DNA integrity was verified by agarose gel electrophoresis, and concentration was quantified using a Qubit2.0 Fluorometer (Thermo Scientific). Sequencing libraries were prepared using the NEBNext Ultra^TM^ DNA Library Prep Kit (New England Biolabs). Whole genome sequencing was performed using both the Nanopore PromethION platform and the Illumina NovaSeq PE150 system (Beijing Novogene Bioinformatics Technology Co., Ltd., Beijing, China). Low‐quality reads were removed using with fastq,^[^ ^63^, ^64^ ^]^ and clean reads were assembled with SPAdes (v3.13.1). Bioinformatic analyses focused on KEGG Orthology.^[^ ^65^ ^]^ A genome overview with the annotation information was visualized using Circos.^[^ ^66^ ^]^ The PSE14 genome has been deposited in the NCBI BioProject database under accession number PRJNA914510.

Mutants for the *iaaM* and *cheA* were generated based on the PSE14 genome sequence using the primers listed in Table S3 (Supporting Information). The suicide vector pRE112 was used to construct PSE14 auxin biosynthesis mutants, as previously described.^[^ ^67^ ^]^ Genomic regions upstream and downstream of *iaaM* and *cheA* were amplified from PSE14. These segments were joined via overlapping PCR, and inserted into the *p*RE112 vector using the *XbaI* restriction site. Recombinant plasmids were first transformed into *E. coli* MC1061 and then into *E. coli* S17‐1. The target DNA segment for complementation was amplified and ligated into the pBBRMCS1 vector at the *XbaI* restriction site. The resulting recombinant plasmid was transformed into the Δ*cheA* or Δ*iaaM* strains by electroporation to generate corresponding complemented strains. Primers used for gene deletion and complemented strain construction were provided in Table S3 (Supporting Information).

### 2.14 Construction of OsCHS1‐Transgenic Rice Plants

For the generation of *OsCHS1* overexpression lines, the open reading frame of *OsCHS1* (*Os11g32650*) was amplified from rice (cv. Nipponbare) using primers listed in Table S3 (Supporting Information). The amplified fragment was ligated into the *p*BWA(V)HS vector and transformed into diploid rice. Total RNA was extracted using TRIzol reagent (Invitrogen). Expression of *OsCHS1* and *OsActin* were analyzed by real‐time quantitative RT‐PCR using the primers in Table S3 (Supporting Information) and the protocol established by Xu et al.^[^ ^56^ ^]^

### 2.15 Quantitative Real‐Time PCR Assay in the Presence of Flavonoids

A single colony of the PSE14 strain was cultured in CFC *Pseudomonas* semi‐selective medium with or without 100 µM apigenin or luteolin at 30 °C while shaking at 200 rpm for 8 h. Total RNA was extracted using the RNeasy Protect Bacteria Mini Kit (Qiagen), in accordance with the manufacturer's protocol. The genes relative expression using the primers in Table S3 (Supporting Information) and the protocol established by Xu et al.^[^ ^56^ ^]^ The *Pseudomonas rpoD* gene served as the internal control.^[^ ^68^ ^]^ Three biological replicates of each sample were used for the assay.

### 2.16 Chemotaxis Assay

The chemotaxis assay was performed using the method of Ling et al.^[^ ^69^] A 200 µL pipette tip served as a chamber containing 100 µL of *Pseudomonas* suspension (OD600 = 0.1) prepared in PBS. A 2 cm 25‐gauge needle (Becton Dickinson) connected to a 1 mL tuberculin syringe was used as the chemotaxis capillary. The syringe was filled with 200 µL of either apigenin or luteolin (at a concentration of 100 µM). After 2 h of incubation at room temperature, the capillary was removed from the *Pseudomonas* suspension. Its content were diluted and plated on CFC *Pseudomonas* semi‐selective medium to quantify colony‐forming units. Three replicates were selected for each experimental measurement, and experiments were repeated twice.

### 2.17 Rhizosheath Formation with P. Nitroreducens (PSE14), Mutant and Complementary Strains Inoculation

Sixty grams of sterilized soil was placed in a 200 mL container, and inoculated with 2 mL of PSE14, mutant and complementary strains, respectively. After inoculation 2 weeks, the number of stains in the soil was determined. Half of the seedlings were inoculated with the bacterial suspension at a concentration of 10^8^ cells g^−1^ soil; and the uninoculated seedlings were treated with sterilized double‐distilled water as a control.^[^ ^67^ ^]^ Next, the seedlings were subjected to the SD treatment described above (Figure S1C, Supporting Information). Total root length, root hair length, rhizosheath soil weight and WUE were measured as described above. Three replicates of the rice plants were selected for each experimental measurement, and experiments were repeated twice.

### 2.18 Effect of P. Nitroreducens on Rice Growth Under the Field Conditions

Field experiments were conducted at a controlled experimental station in Yangzhong Town, Fujian Province (N26°17′, E118°29′). The experimental plots were fertilized with 100 kg ha^−1^ N, 90 kg ha^−1^ P_2_O_5_ and 150 kg ha^−1^ K_2_O to prevent nutrient deficiencies. For inoculation, a mixed bacterial suspension (in 50 µM PBS, pH 7.0, OD600 = 1.0) was directly applied to rice seedlings and soil. Control plants received an equal volume of PBS. Seedlings were transplanted with a spacing of 25 × 20 cm. Fungicides and insecticides were applied to manage pests and diseases, and weeds were manually removed. The SD treatment was administered as previously described.^[^ ^70^ ^]^ At plant maturity, total tiller number, shoot dry weight and grain yield were recorded.

### 2.19 Statistical Analysis

Graphical representations were generated using Prism v8.0.2 (GraphPad Software, Inc., La Jolla, CA, USA). Phylogenetic analysis of *Pseudomonas* sequences was performed using Molecular Evolutionary Genetics Analysis v7.0 (MEGA7). Statistical comparisons among treatments were performed using one‐way ANOVA followed by Tukey's HSD (two‐sided). For pairwise comparisons, the two‐tailed Student's *t* test was applied. Permutational multivariate analysis of variance (PERMANOVA) was used to assess the differences in microbial community structure. Differences in alpha‐diversity (based on Chao 1 and Shannon indices) were evaluated using the Wilcoxon rank‐sum test. For principal coordinates analysis (PCoA), permutational multivariate analysis of variance (PERMANOVA) was performed using the *Adonis* function with 999 permutations based on Bray‐Curtis distances. The Kruskal‐Wallis sum‐rank test was used to identify the features with significantly different abundances among assigned bacterial families in the LEfSe analysis.

## 3 Results

### 3.1 Tetraploid Rice Showed Increased Water Use Efficiency and a Large Rhizosheath Under Soil Drying

**Figure 1:**
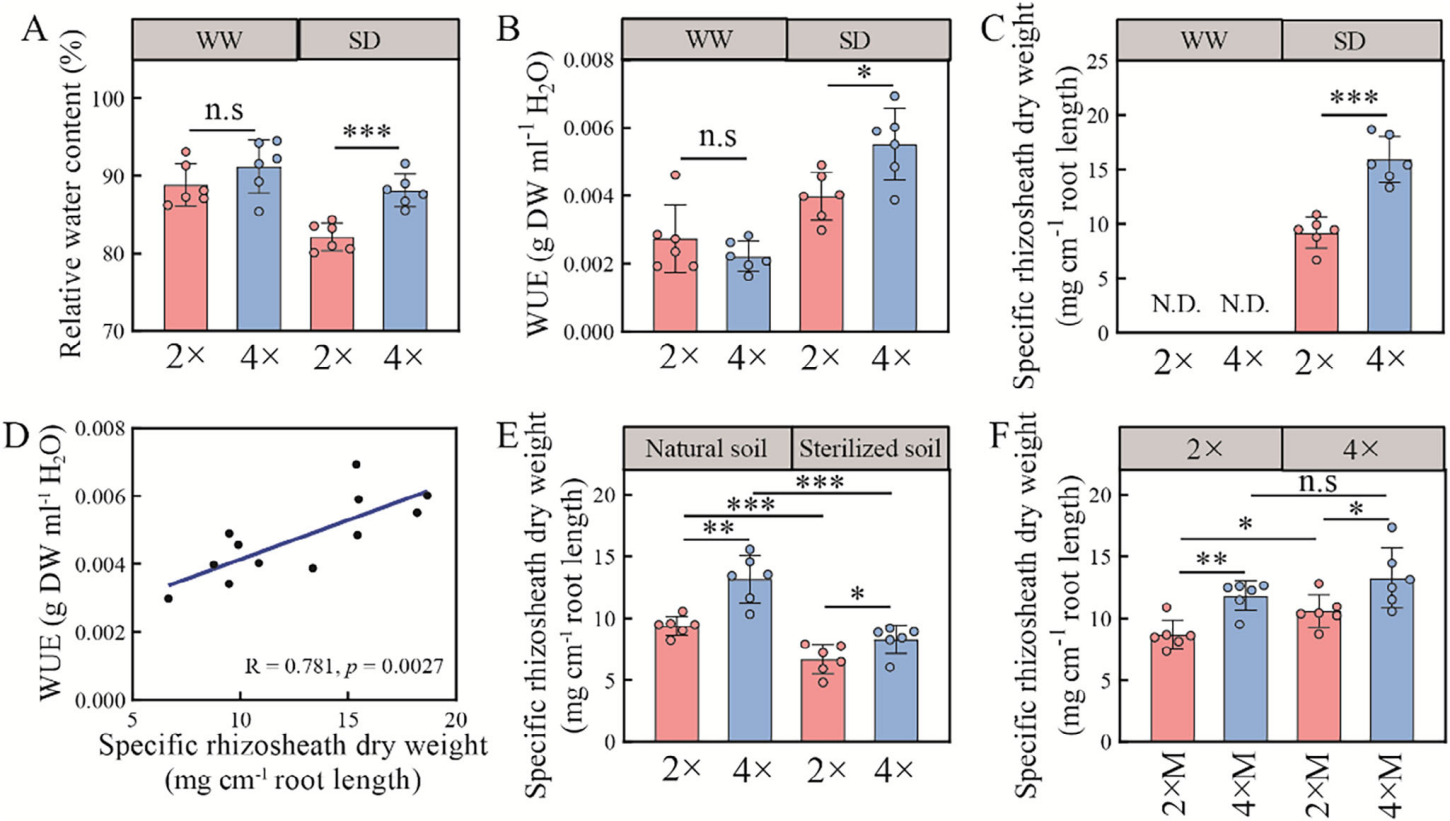
Polyploidy enhances rice water use efficiency through microbiota‐modulated rhizosheath formation under soil drying (SD). A,B) The relative water content of rice leaf (A) and water‐use efficiency (WUE, B) in diploid (2 ×) and tetraploid (4 ×) rice under well‐watered (WW) and SD conditions. C) Rhizosheath formation of diploid (2 ×) and tetraploid (4 ×) rice under WW and SD conditions. N.D. indicates that rhizosheath dry weight was not detectable. D) Correlation between rhizosheath formation and WUE under SD conditions. E) Rhizosheath formation in diploid (2 ×) and tetraploid (4 ×) rice grown under SD, with or without prior soil sterilisation. F) Rhizosheath formation in diploid (2 ×) rice and tetraploid (4 ×) rice when inoculated with the rhizosheath microbiota of diploid rice (2 × M) or the rhizosheath microbiota of tetraploid rice (4 × M). All data are means ± SE (*n* = 6). Statistical significances were evaluated using a two‐tailed Student's *t*‐test. *p <0.05; **p <0.01; ***p <0.001; n.s, not significant.

To test the key morphological traits in diploid (2 ×) and tetraploid rice (4 ×), rice seedlings were grown. No significant differences were observed in plant height, shoot/root fresh weight or shoot/root dry weight between diploid and tetraploid rice (Figure S2, Supporting Information). However, tetraplid rice exhibited 24.6% greater total root length, 140% longer root hair length and 44.0% higher root hair density than diploid rice (Figure S2, Supporting Information), which suggested that root hair may contribute to drought tolerance. Further, the rice growth and metabolic status were measured to offer a more comprehensive understanding of overall physiological condition. The net rate of CO_2_ assimilation of tetraploid rice was 22.2% higher than diploid rice (Figure S3, Supporting Information). No significant differences were detected in the free proline or MDA contents between diploid and tetraploid rice (Figure S3, Supporting Information). Under SD, the primary root elongation rate, root hair length and root hair density of tetraploid rice was increased by 580%, 74.7% and 59.2%, respectively, relative to diploid rice (Figure S4, Supporting Information), which suggests that tetraploid rice may enhance drought tolerance via more extensive root system. The information provided a more complete understanding of the physiological mechanisms involved in stress resilience for polyploidy rice. With respect to drought tolerance, tetraploid rice maintained 7.3% higher leaf water content under SD, and 2.6% higher content under WW, compared with diploid rice (**Figure** **1**A). Under SD, tetraploid rice exhibited 39% greater WUE and 19% higher iWUE than diploid rice; no significant differences were observed under WW (Figures 1B; Figure S5A, Supporting Information). To explore whether rhizosheath involved in the drought tolerance in polyploidy rice for, rhizosheath formation was determined in tetraploid and diploid rice under drought conditions. No rhizosheath soil was attached to roots under WW conditions (Figure 1C). Under SD, the specific rhizosheath soil weight of tetraploid rice was 73% higher than that of diploid rice (Figure 1C). A significant and positive linear correlation was observed between WUE (R^2^ = 0.781, *p* = 0.0027) or iWUE (R^2^ = 0.342, *p* = 0.00458) and specific rhizosheath soil dry weight under SD (Figure 1D; Figure S5B, Supporting Information), which showed that rhizosheath formation contributed to rice drought tolerance. The rhizosheath soil water contents of diploid and tetraploid rice were 37.8% and 57.7% higher, respectively, than that of bulk soil under SD (Figure S5C, Supporting Information). Compared with diploid rice, the rhizosheath soil water content in tetraploid rice was increased by 14.5% under SD (Figure S5C,D, Supporting Information). These results showed that tetraploid rice enhanced rhizosheath formation and water‐use efficiency under SD.

### 3.2 Microbiota Associated with Rhizosheath Formation in Tetraploid Rice Under Soil Drying

To evaluate the effect of soil microbiota on rhizosheath formation, rice plants were cultivated in natural and sterilized soil under SD. Olsen‐P concentration were not significantly different between the two soil (Figure S6A, Supporting Information). Under SD and in natural soil, tetraploid rice exhibited a 40% higher specific rhizosheath soil weight compared with diploid rice; in sterilized soil, this value was 24% higher (Figure 1E). These results suggest that soil bacteria enhance rhizosheath formation in tetraploid rice under SD. Root hairs of tetraploid rice were 24% and 18% longer than those of diploid rice in natural and sterilized soil, respectively, under SD (Figure S6B, Supporting Information). To validate the role of microbiota in rhizosheath formation, rhizosheath microbiota collected from both diploid and tetraploid rice were separately inoculated onto diploid and tetraploid rice seedlings (Figure 1F). In diploid rice inoculated with the rhizosheath microbiota from tetraploid rice (4 × M), rhizosheath formation increased by 36% relative to those inoculated with microbiota from diploid rice (2 × M) under SD (Figure 1F). The specific rhizosheath soil weight of 4 × M‐inoculated diploid rice was similar to that of tetraploid rice inoculated with its own microbiota. Root hair length followed the same trend as specific rhizosheath soil weight (Figure S6C, Supporting Information). The results showed that microbiota contribute to the enhanced rhizosheath formation observed in tetraploid rice under SD.

### 3.3 Rice Bacterial Composition Among Rhizocompartments

**Figure 2:**
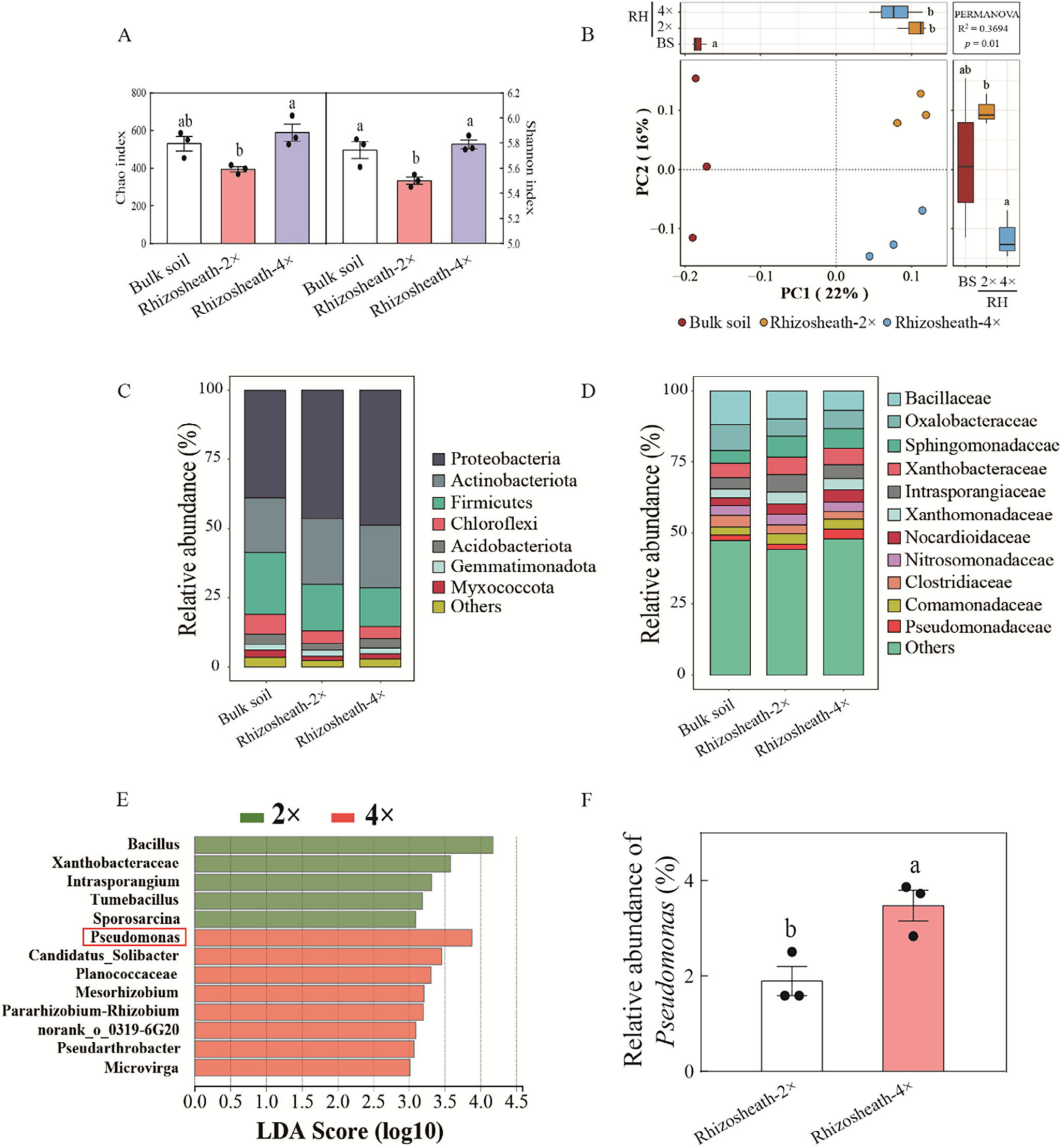
The relative abundance of genus *Pseudomonas* is promoted in tetraploid rice relative to diploid rice under soil drying (SD). A) The alpha‐diversity (based on Chao and Shannon index) of diploid (2 ×) and tetraploid (4 ×) rice under SD. Different letters represent significant difference among the treatments at *P* <0.05 (Wilcoxon rank‐sum test). B) Principal‐coordinate analysis (PCoA, based on Bray‐Curtis distance) showing the difference in the composition of bacterial microbiomes in the bulk soil, rhizosheath soil of diploid (Rhizosheath‐2 ×) and tetraploid (Rhizosheath‐4 ×) rice under SD. PERMANOVA (Adonis function, 999 permutations) was used to test the difference in the composition of bacterial microbiomes in PCoA. C,D,) The relative abundances of bacteria taxa at the phylum (C) and family (D) levels in the rhizosheath soil of diploid (2 ×) and tetraploid (4 ×) rice under SD. E) Linear discriminant analysis (LDA) of bacterial taxa at the genus level showing significant difference in the abundance between the rhizosheath soil of diploid (2 ×) and tetraploid (4 ×) rice (LDA score ≥ 3.0) under SD. F) The relative abundance of the *Pseudomonas* genus in the rhizosheath soil of diploid (2 ×) and tetraploid (4 ×) rice, as well as in bulk soil under SD. Data are means ± SE (*n* = 3). Bars with different letters are significantly among different treatments at *p* <0.05 (one‐way ANOVA, Tukey's HSD, two‐sided).

To characterize microbial community structures influenced by polyploidy, we built the 16S rRNA amplicon libraries for bulk soil, phyllosphere, rhizosphere, rhizosheath and root endosphere samples from diploid and tetraploid rice under SD and WW conditions. A total of 2,318,524 high‐quality sequences were obtained from 42 samples (average: 55,202; range: 31,599‐74,290; Table S4, Supporting Information). Rarefaction curves plateaued, indicating that the sequencing depth was sufficient to capture microbial diversity (Figure S7, Supporting Information). Under SD, alpha‐diversity in rhizosheath soil was significantly higher among tetraploid rice than among diploid rice (**Figure** **2**A). In contrast, no significant differences were observed in Chao 1 and Shannon diversity indices for rhizosphere soil under WW (Figure S8A, Supporting Information). Principal coordinate analysis (PCoA, based on Bray‐Curtis) revealed that polyploidy significantly influenced bacterial community composition in rhizosheath soil under SD and in rhizosphere soil under WW (Figure 2B; Figure S8B, Supporting Information). However, no significant differences were detected in bacterial communities of the root endosphere or phyllosphere between diploid and tetraploid rice (Figure S9,B, Supporting Information).

The dominant phyla in rhizosheath soil of diploid and tetraploid rice under both SD and WW included Proteobacteria, Actinobacteria, Firmicutes, Chloroflexi, Acidobacteriota, Gemmatimonadota and Myxococcota (Figure 2C). At the family level, Paenibacillaceae (relative abundance 3.49%), Rhizobiaceae (1.82%) and Solibacteraceae (1.32%) were more abundant in tetraploid rice than in diploid rice under SD (Figure 2D). Under WW, Haliangiaceae (0.40%), Acidothermaceae (0.37%) and Frankiaceae (0.36%) were significantly more abundant in the rhizosphere of tetraploid rice relative to diploid rice (Figure S8C, D, Supporting Information). There was no significant change in the abundance of Pseudomonadaceae in rhizosphere soil between diploid and tetraploid rice under WW (Figure S8C,D, Supporting Information). The abundance of Xanthomonadaceae and Oxalobacteraceae in the root endosphere under SD significantly differed (Figure S9D,F, Supporting Information). There was no significant change in Pseudomonadaceae abundance between diploid and tetraploid rice in phyllosphere or root endosphere under either SD or WW (Figure S9C,E,F, Supporting Information).

Given that differences between diploid and tetraploid rice were primarily observed in the rhizosheath and rhizosphere microbiomes (Figure 2B; Figure S8B, Supporting Information), we focused on these aspects. A LEfSe approach was used to identify taxa associated with rhizosheath formation (Figure 2E). In rhizosheath soil under SD, *Pseudomonas* (LDA score, 3.88) was specifically enriched in tetraploid rice relative to diploid rice (Figure 2E). The abundance of the genus *Pseudomonas* was 83.4% higher in the rhizosheath soil of tetraploid rice compared with diploid rice under SD, whereas no significant difference was detected under WW (Figure 2F; Figure S10A, Supporting Information). The result was confirmed by colony counting (Figure S10B, Supporting Information). Together, these results indicated a correlation between increased rhizosheath formation and the enhanced abundance of *Pseudomonas* in tetraploid rice. Thus, we focus to explore the role of *Pseudomonas*, isolated from rhizosheath of tereaploid rice, for rice rhizosheath formation.

### 3.4 Pseudomonas was Recruited by Root Flavonoids Under Soil Drying

**Figure 3:**
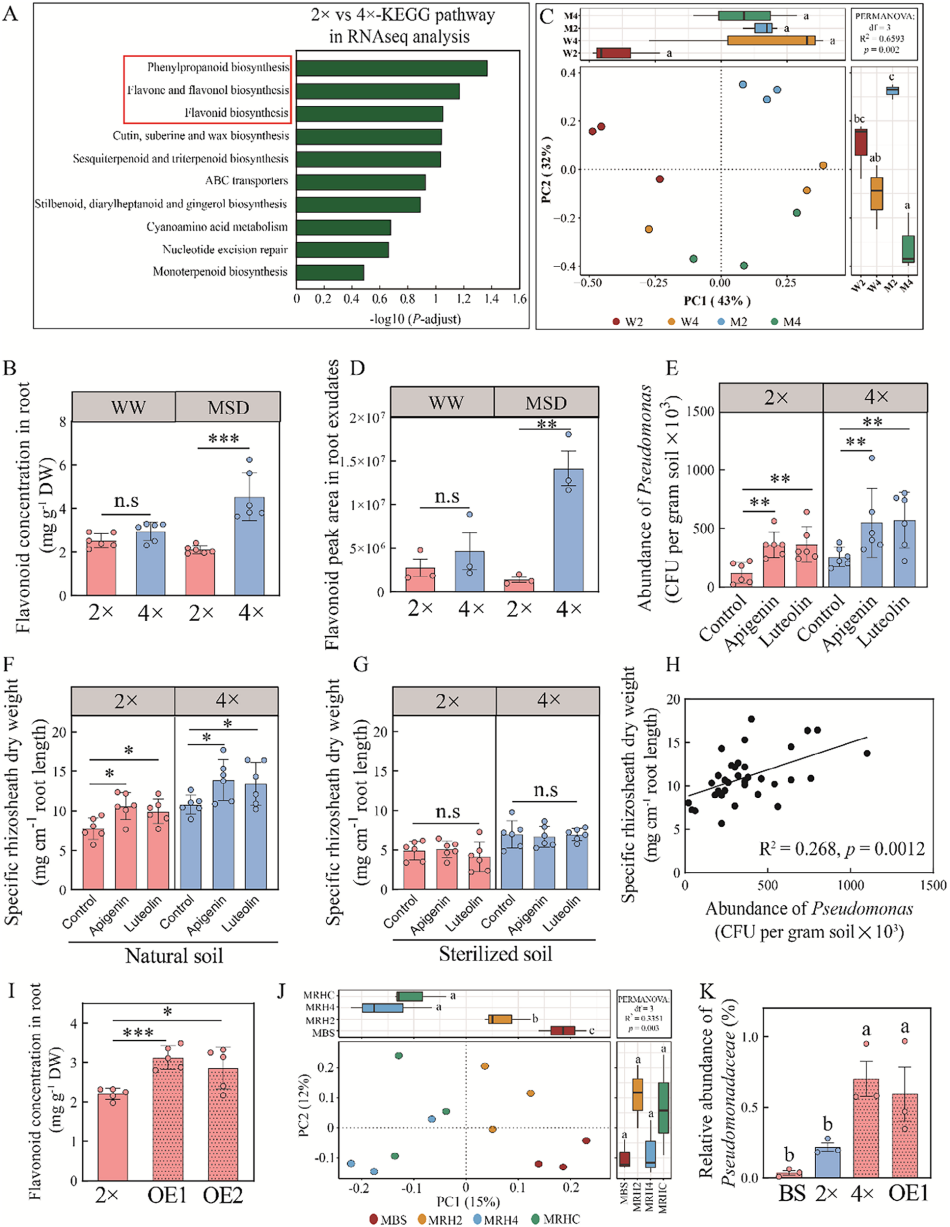
The increased *Pseudomonas* abundance and rhizosheath formation in tetraploid rice are associated with the flavonoids of root exudates in tetraploid rice under soil drying (SD). A) KEGG pathways of differentially expressed genes (DEGs) between diploid (2 ×) and tetraploid (4 ×) rice under SD. DEGs were identified by gene‐expression‐level analysis with fold change values ≥ 2. B) Flavonoid contents in diploid (2 ×) and tetraploid (4 ×) rice root under the well‐watered (WW) and SD conditions. Data are the means ± SE (*n* = 6). Differences were evaluated using the two‐tailed Student's *t* test (***p <0.001; n.s not significant). C) Principal component analysis of samples (W2, W4, M2 and M4) based on flavonoid metabolite analysis. W2, W4, M2 and M4 represent diploid (2 ×) and tetraploid (4 ×) rice under WW and SD conditions, respectively. D) Abundance (cumulative peak area) of flavonoids in diploid (2 ×) and tetraploid (4 ×) rice exudates under WW or SD based on flavonoid metabolite analysis. Data are the means ± SE (*n* = 3). Differences were evaluated using the two‐tailed Student's *t* test (**p <0.01; n.s not significant). E) Abundance of cultivable *Pseudomonas* in rhizosheath of diploid (2 ×) and tetraploid (4 ×) rice with addition of exogenously applied flavonoid under SD. Data are the means ± SE (*n* = 6). Differences were evaluated using the two‐tailed Student's *t* test (*p <0.05; **p <0.01). F,G) Effects of exogenously applied flavonoids on rhizosheath formation of diploid (2 ×) and tetraploid (4 ×) rice in sterilized soil (E) and natural soil (F) under SD. Data are the means ± SE (*n* = 6). Differences were evaluated using the two‐tailed Student's *t* test (*p <0.05; n.s not significant). H) Correlation between rhizosheath formation and abundance of *Pseudomonas* in the rhizosheath soil of diploid (2 ×) and tetraploid (4 ×) rice with the addition of exogenously applied flavonoid types under SD. *p* value was calculated based on two‐sided *t* test. I) Root flavonoid concentration of wild‐type and *OsCHS1*‐overexpressing lines (OE1 and OE2). Data are the means ± SD (*n* = 3). Differences were evaluated using the two‐tailed Student's *t* test (*p <0.05, ***p <0.001). J) Principal‐coordinate analysis (PCoA, based on Bray‐Curtis distance) of bacterial microbiomes in the bulk soil, rhizosheath soil of diploid (2 ×), tetraploid (4 ×) rice and *OsCHS1*‐overexpressing line OE1 under SD. PERMANOVA (Adonis function, 999 permutations) was used to test differences in the composition of bacterial microbiomes in PCoA. MBS, bulk soil; MRH2, rhizosheath soil of diploid rice under SD; MRH4, rhizosheath soil of tetraploid rice under SD; MRHC, rhizosheath soil of *OsCHS1*‐overexpressing line OE1 under SD. K) The relative abundance of Pseudomonadaceae in the rhizosheath soil of bulk soil (BS), diploid (2 ×), tetraploid (4 ×) and *OsCHS1*‐overexpressing line OE1 rice under SD conditions. Data are the means ± SD (*n* = 3). Bars with different letters are significantly among different treatments at *p* <0.05 (one‐way ANOVA, Tukey's HSD, two‐sided).

To further investigate the effect of tetraploid rice on rhizosheath microbiota, RNA‐seq and metabolomic analyses were performed. We obtained 4.72‐5.47 × 10^7^ clean mRNA reads per sample. Under SD, tetraploid rice exhibited 1501 upregulated and 2902 downregulated DEGs relative to diploid rice (Figure S11, Supporting Information). KEGG pathway enrichment revealed that flavonoid biosynthesis was significantly enriched in tetraploid rice compared with diploid rice under SD (**Figure** **3**A, Table S5, Supporting Information). Metabolomic analysis revealed 794 upregulated and 841 downregulated metabolites in tetraploid rice relative to diploid rice under SD (Figure S12A, Supporting Information). KEGG analysis identified enrichment in phenylpropanoid and flavonoid biosynthesis pathways among the differentially abundant metabolites (Figure S12B; Table S6, Supporting Information). The relative abundances of organic and phenolic acids were also increased in tetraploid rice under SD (Table S6, Supporting Information), which suggested that these compounds play roles in plant‐microbe interactions and polyploidy‐mediated stress responses. As the flavonoid related pathways were identified in both RNA‐seq and metabolomic analyses, we hypothesized that the flavonoids were involved in shifting rhizosheath micribiota. The concentration of root flavonoid in tetraploid rice was 115.5% higher than in diploid rice under SD; no significant difference was observed under WW (Figure 3B). To further explore the flavonoid shifting rice rhizosheath microbiome, flavonoid metabolites in root exudates was performed. Flavonoid metabolites profiles were significantly different between tetraploid and diploid rice under SD (Figure 3C). Tetraploid rice secreted significantly higher levels of flavonoids than diploid rice under SD, but there was no significant difference under WW (Figure 3D). Of the 110 flavonoid metabolites, 42 (including apigenin 7‐methyl ether, apigenin‐6‐C‐arabinoside‐8‐C‐xyloside, apigenin‐6‐C‐xyloside‐8‐C‐arabinoside, apigenin‐6,8‐di‐C‐arabinoside, luteolin‐8‐C‐arabinoside, luteolin‐6‐C‐(5''‐glucuronyl)xyloside and luteolin‐6‐C‐arabinoside‐7‐O‐glucoside) significantly differed between diploid and tetraploid rice under SD (Figure S13A, Table S7, Supporting Information). These flavonoid metabolites are primarily involved in “Flavonoid biosynthesis”, “Flavone and flavonol biosynthesis” and “Biosynthesis of secondary metabolites” pathways (Figure S13B, Supporting Information).

Given that apigenin‐ and luteolin‐related flavonoids were the different flavonoid‐compounds between diploid and tetraploid rice under SD, we applied apigenin and luteolin in natural and sterilized soil to evaluate their effects on *Pseudomonas* recruitment under SD (Figure 3E–G). In diploid rice under SD, the abundances of culturable *Pseudomonas* in rhizosheath soil increased by 157% and 174%, respectively, after treatment with apigenin and luteolin (Figure 3E). Corresponding increases in tetraploid rice were 1.49‐ and 1.99‐fold, respectively (Figure 3E). In natural soil, rhizosheath formation and root hair length in tetraploid rice increased by 27.8–36.5% and 12.4–13.4%, respectively, compared with the untreated control under SD (Figure 3F; Figure S14A, Supporting Information). In diploid rice, the corresponding increases were 24.4–29.1% and 16.9–22.5% (Figure 3F). However, these enhancements were not observed in sterilized soil (Figure 3G; Figure S14B, Supporting Information). A significant and positive linear correlation was detected between rhizosheath formation and *Pseudomonas* abundance under SD (R^2^ = 0.27, *p* <0.0012; Figure 3H).

To confirm the role of flavonoids in the increased *Pseudomonas* abundance, we overexpressed *OsCHS1*, a key gene involved in flavonoid biosynthesis, in diploid rice. Under SD conditions, the relative expression of *OsCHS1* in tetraploid rice roots increased by 2.09‐fold compared with diploid rice (Figure S15A, Supporting Information). In the overexpressing lines (OE1 and OE2), *OsCHS1* relative expression in roots was 208‐ and 206‐fold higher, respectively, than in diploid rice (Figure S15B, Supporting Information). The roots flavonoid concentration in OE1 and OE2 was elevated by 42.0% and 29.7%, respectively, compared with diploid rice (Figure 3I). 16S rRNA sequencing was conducted for verifying the role of flavonoids in *Pseudomonas* abundance. PCoA revealed a significant difference in the bacterial community composition of rhizosheath between OE1 and diploid rice; but no difference was observed between OE1 and tetraploid rice (Figure 3J). The relative abundances of Pseudomonadaceae in the rhizosheath soil of OE1 and tetraploid rice were by 2.7‐ and 3.2‐fold higher, respectively, compared with diploid rice under SD (Figure 3K). No significant difference was found between OE1 and tetraploid rice in this regard (Figure 3K). Colony counting confirmed that the abundance of culturable *Pseudomonas* in OE1 rhizosheath was significantly higher than in diploid rice under SD (Figure S15C, Supporting Information). Furthermore, the changes in *Pseudomonas* populations and flavonoid levels were measured in pot conditions. The root flavonoid concentration of tetraploid rice after 21, 28 and 35 days of SD treatments was increased by 37%, 44.6% and 46.2% relative to diploid rice (Figure S16A, Supporting Information). Compared with diploid rice, the abundance of culturable *Pseudomonas* in tetrploid rice after 21, 28 and 35 days of SD treatments was 76.9%, 79.8% and 85.5% higher than diploid rice under SD (Figure S16B, Supporting Information). A significant and positive linear correlation was detected between root flavonoid concentration and *Pseudomonas* abundance under SD (R^2^ = 0.6983, *p* <0.0001; Figure S16C, Supporting Information). Altogether, the results showed that tetraploid rice can recruit *Pseudomonas* under SD through synthesizing flavonoids in the roots.

### 3.5 Histidine Kinase is Involved in Flavonoid‐Induced Pseudomonas Recruitment

**Figure 4:**
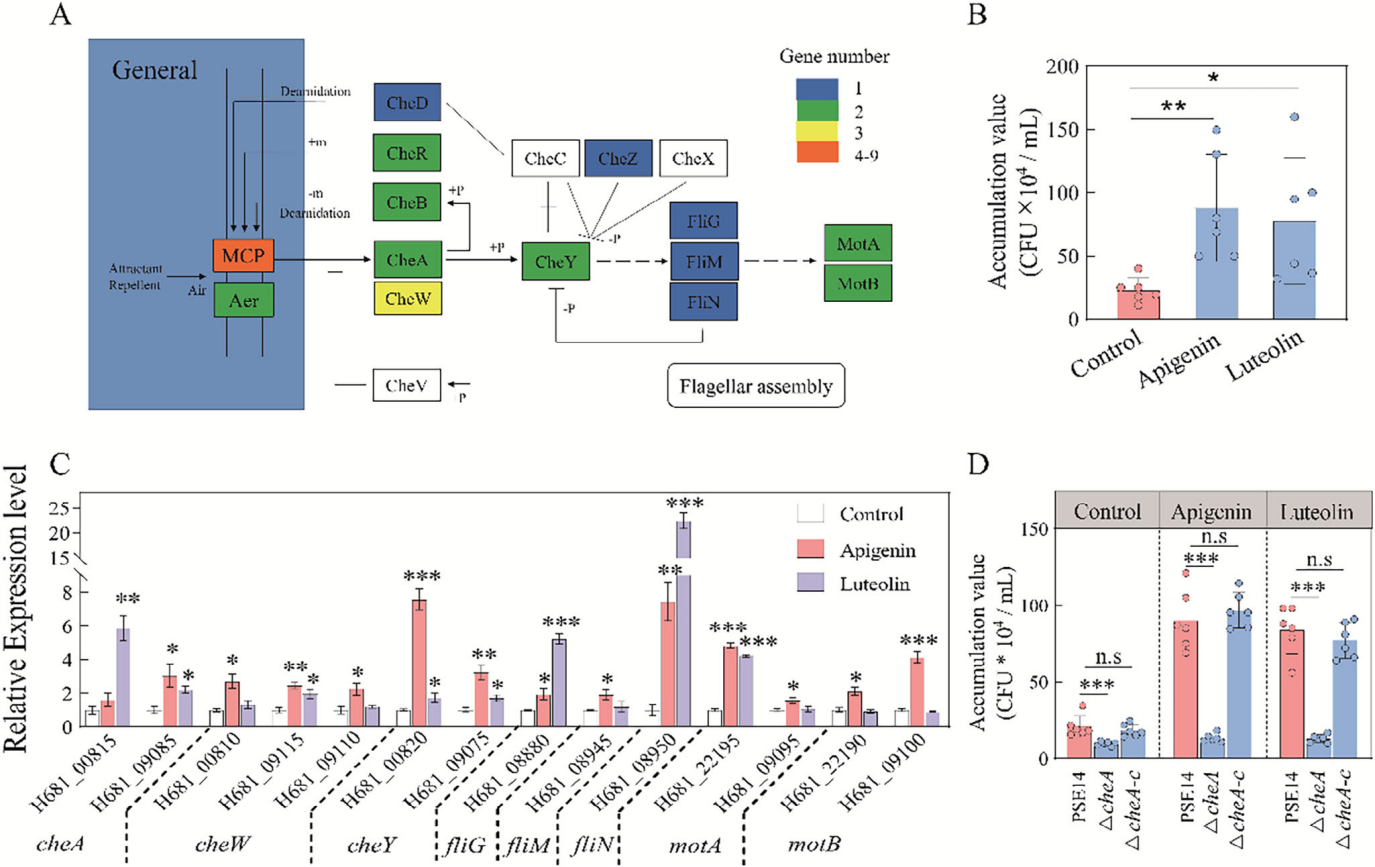
*Pseudomonas nitroreducens* strain PSE14 displays flavonoid‐induced chemotaxis. A) Bacterial chemotaxis pathway in the strain PSE14 genome based on KEGG analysis. Different colors indicate gene numbers in the strain PSE14 genome. Aer: Chemoreceptor for chemotaxis; CheR and MCP: Methyl‐accepting chemotaxis proteins; CheY, CheW, CheV, CheD and CheA: Chemotaxis proteins; CheB, Chemotaxis response regulator protein‐glutamate methylesterase; CheZ: Protein phosphatase; MotA, MotB: Flagellar motor protein. FliM, FliG and FliN: Flagellar motor switch proteins. B) Chemotactic response of PSE14 towards apigenin and luteolin. Accumulation of bacteria in the capillaries was calculated as the average from the CFUs. PBS buffer is used as the control. C) Quantitative RT‐PCR results of chemotaxis‐related genes based on KEGG analysis. D) The chemotaxis of PSE14, Δ*cheA* mutant and complemented strain Δ*cheA‐c* in response to apigenin and luteolin. Data are the means ± SD (*n* = 6 for B and D, *n* = 3 for C). Asterisks indicate significant difference among different treatments at *p* <0.05 (two‐sided Student's *t* test). *P <0.05; **P <0.01; ***P <0.001; n.s, not significant.

To determine whether the enrichment of *Pseudomonas* contributes to rhizosheath formation under SD, 54 *Pseudomonas* strains were randomly isolated from the rhizosheath soil of tetraploid rice under SD (Table S8, Supporting Information). Based on 16S rRNA gene sequencing, these strains were categorized into 12 distinct phylogenic groups (Figure S13A, Supporting Information). *P. nitroreducens* strain PSE14 showed 99% sequence identity with the most responsive *Pseudomonas* ASV, i.e. ASV1 (Figure S17A, Supporting Information). To confirm whether flavonoids recruit PSE14 through chemotaxis, we performed whole genome sequencing of PSE14. The PSE14 genome was approximately 5,977,163 bp with a GC content of 65.2% (Figure S17B, Supporting Information). Genes associated with bacterial chemotaxis and flagellar assembly pathways were identified (**Figure** **4**A; Figure S18, Tables S9,S10, Supporting Information). Moreover, PSE14 demonstrated strong chemotactic responses toward apigenin and luteolin relative to the control (Figure 4B). Upon the addition of apigenin and luteolin, the expression of chemotaxis‐associated genes (*cheA*, *cheW*, *cheY*) was increased by 63%–489% compared with control (Figure 4C). The expression of flagellum‐associated genes (*fliG*, *fliM*, *fliN*, *motA* and *motB*) in the addition of apigenin and luteolin were 19%‐2147% higher than that in control (Figure 4C). The strain with addition of luteolin showed 8% and 10% reduction expression in *motA* and *motB*, relative to control (Figure 4C).

To further assess whether histidine kinase is necessary for flavonoid‐induced chemotaxis, a *cheA* mutant and a complemented strain were constructed in the PSE14 background (Figure S19A, Supporting Information). Compared with wild‐type PSE14, chemotactic activity in the Δ*cheA* mutant was reduced by 53.5%, 85.1% and 84.5% under control, apigenin and luteolin treatments, respectively (Figure 4D). There was no significant difference of chemotaxis between PSE14 and complemented strain Δ*cheA‐c* with or without addition of flavonoids (Figure 4D). Although the addition of apigenin and luteolin increased the chemotactic response of PSE14 by 4.3‐ and 4.0‐fold, respectively, over the control, no significant difference was observed in the Δ*cheA* mutant (Figure 4D). In addition, there was no significant difference of relative expression of flagellar genes (*fliG*, *fliM* and *fliN*) between PSE14 and Δ*cheA* (Figure S20, Supporting Information). These results collectively showed that transcriptional regulation of chemotaxis related genes, particularly *cheA*, is crucial for the flavonoid‐mediated recruitment of *Pseudomonas* under SD.

### 3.6 Function of P. Nitroreducens in Rice Rhizosheath Formation

**Figure 5:**
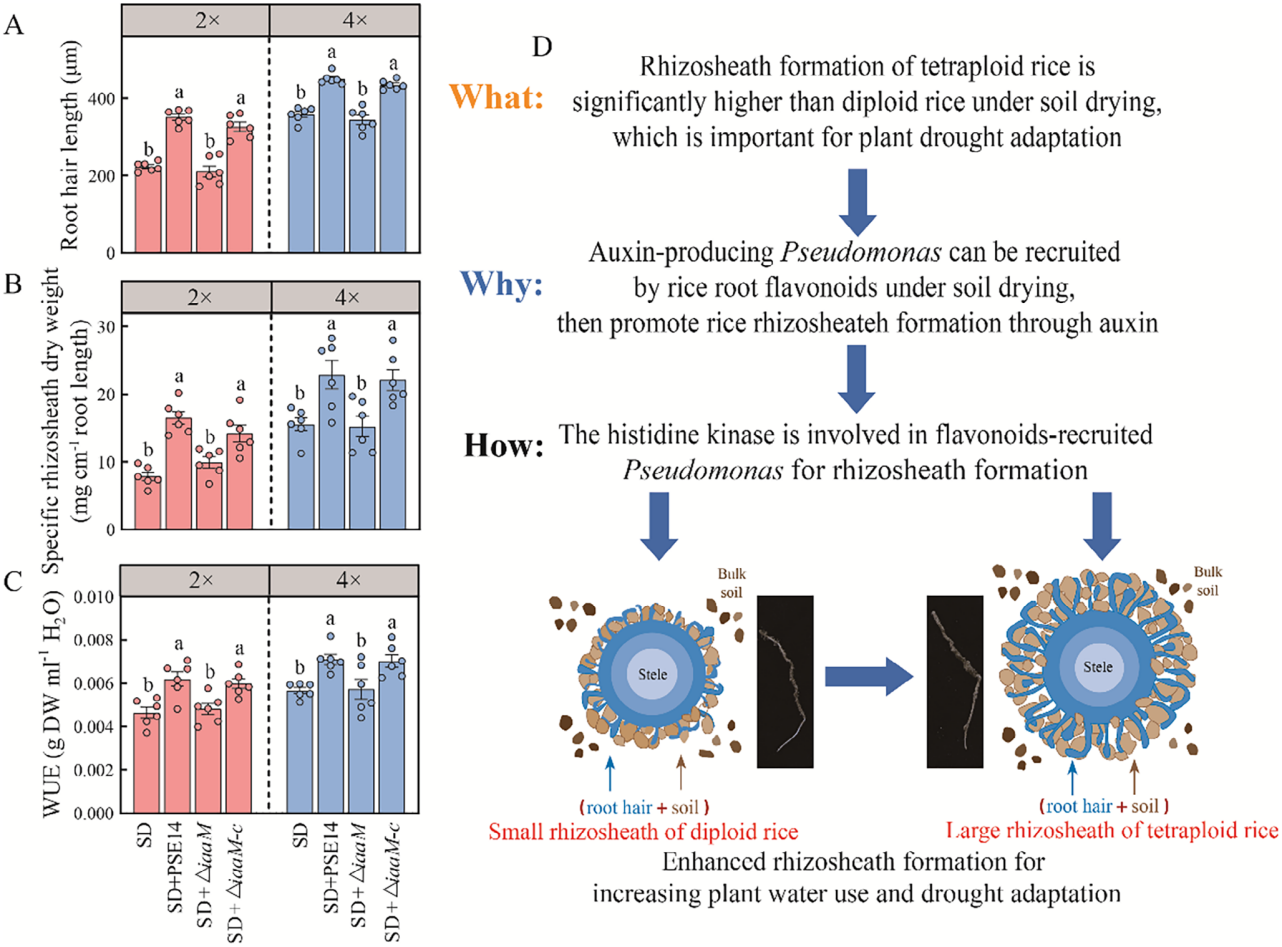
Rhizosheath formation of tetraploid rice is enhanced via *Pseudomonas nitroreducens* strain PSE14 inoculation through auxin production under soil drying (SD). A–C) Root hair length (A), rhizosheath formation (B) and water‐use efficiency (WUE) (C) of diploid (2 ×) and tetraploid (4 ×) rice with no inoculation (SD), PSE14 inoculation (SD + PSE14), Δ*iaaM* inoculation (SD + Δ*iaaM*) and Δ*iaaM‐c* inoculation (SD + Δ*iaaM‐c*) under SD. Data are the means ± SE (*n* = 6). Bars with different letters indicate significant differences among treatments at *p* <0.05 (one‐way ANOVA, Tukey's HSD, two‐sided). D) Proposed model for increased rhizosheath formation in tetraploid rice under SD. Briefly, the high content of flavonoids in tetraploid rice resulted in the enrichment of *Pseudomonas* abundance under soil drying. Consequently, more *Pseudomonas* ensures auxin production, which promoted root hair growth and rhizosheath formation under SD.

Several genes associated with indole‐3‐acetic acid (IAA) biosynthesis were identified in the PSE14 genome (Table S11, Supporting Information), and PSE14 was capable of producing IAA (Figure S18C, Supporting Information). There was no significant difference in root IAA concentration between diploid and tetraploid rice (Figure S21, Supporting Information), thus implicating PSE14 in rice rhizosheath formation. Diploid and tetraploid rice treated with PSE14 and NPA (a polar auxin transport inhibitor) showed significantly reduced root hair length, rhizosheath formation and WUE relative to plants inoculated with PSE14 alone under SD (Figure S22A–C, Supporting Information). To further confirm whether *P. nitroreducens* promotes rhizosheath formation through auxin production, we developed a mutant of the tryptophan‐2‐monooxygenase gene (*iaaM*) in PSE14 along with a complemented stain Δ*iaaM‐*c (Figure S19B, C, Supporting Information). The IAA‐producing capability of Δ*iaaM* significantly decreased by 59.9%, compared with wild‐type PSE14 (Figure S19C, Supporting Information). There was no significant difference of IAA concentration between PSE14 and the complemented stain Δ*iaaM‐*c (Figure S19C, Supporting Information). After inoculation of PSE14, Δ*iaaM* and Δ*iaaM‐*c strains in sterilized soil, we observed no significant differences in abundance of *Pseudomonas* cells (Figure S23, Supporting Information). To explore the effect of bacterial auxin on rice rhizosheath formation, rice plants were inoculated with PSE14, Δ*iaaM* and Δ*iaaM‐*c strains under SD (**Figure** **5**). Compared with the non‐inoculated treatment, root hair length, rhizosheath formation and WUE in diploid rice were increased by 57.4%, 121%, and 33.3%, respectively, after PSE14 inoculation under SD. In tetraploid rice, these increases were 25.8%, 47.1% and 25.4%, respectively (Figure 5A–C). These traits were not significantly altered in either diploid or tetraploid rice inoculated with the Δ*iaaM* relative to non‐inoculated controls (Figure 5A–C). Furthermore, inoculated with PSE14 exhibited 30.6‐66.8% longer root hair, 50.2‐66.5% greater rhizosheath formation and 24.0‐28.9% higher WUE than those inoculated with the Δ*iaaM* mutant in diploid and tetraploid rice (Figure 5A–C). These traits were not significantly changed in either diploid or tetraploid rice inoculated with the PSE14 to Δ*iaaM‐*c (Figure 5A–C). To verify the effect of PSE14 on drought resistant, the field experiments were conducted under SD conditions. Under field conditions, the shoot dry weight, tiller number and grain yield of diploid rice inoculated with PSE14 at maturity were 36.7%, 36% and 35.2% higher, respectively, than those of non‐inoculated controls under SD (Figure S24, Supporting Information). Overall, these results showed that *Pseudomonas* can promote rice rhizosheath formation and enhance drought tolerance in rice through IAA‐dependent mechanism.

## 4 Discussion

### 4.1 Plant Polyploidy Enhances Rice Rhizosheath Formation Under Soil Drying

The present study provides the first evidence that tetraploid rice develops significantly larger rhizosheath under SD than diploid rice (Figure 1C), suggesting role for polyploidy in promoting drought tolerance via rhizosheath formation. Consistent with these findings, tetraploid rice exhibited higher leaf water content and WUE under SD relative to diploid rice (Figure 1A,B; Figure S5A, Supporting Information), in line with the findings of del Pozo & Raminrez‐Parra (2014).^[^ ^31^ ^]^ Furthermore, polyploidy has been shown to enhance drought tolerance by modulating traits such as transpiration rate, photosynthetic efficiency, phonological development and antioxidant levels.^[^ ^71^, ^72^, ^73^ ^]^ In this study, the rhizosheath soil of tetraploid rice exhibited significantly higher water content under SD than that of diploid rice (Figure S5C, Supporting Information), likely due to enhanced water infiltration in the rhizosheath soil.^[^ ^74^ ^]^ The observed positive linear relationship between WUE and rhizosheath formation under SD further suggests that the rhizosheath can be emerged as a valuable breeding target for improving water uptake under drought conditions (Figure 1D; Figure S5B, Supporting Information).^[^ ^6^, ^14^, ^16^ ^]^

### 4.2 Tetraploid Rice Recruits Bacterium Pseudomonas through Flavonoid‐Induced Chemotaxis Under Soil Drying

The phytohormone response pathway, cell proliferation and transpiration rate can explain the improvement of drought resistance in polyploidy.^[^ ^29^, ^30^, ^31^ ^]^ The allotetraploid had an enhanced capacity for rhizobial interactions relative to its diploid ancestors,^[^ ^75^ ^]^ suggesting that the microbiota may be involved in rice drought resistance. In the present study, we explored the contributions of tetraploid rice associated microbiota to rhizosheath formation under soil drying conditions. We found that rhizosheath formation in tetraploid rice was greater than that in diploid rice under SD (Figure 1C). The *Pseudomonas* abundance in tetraploid rice was significantly increased compared with diploid rice (Figure 2F), which suggests the enhanced abundance of *Pseudomonas* may be involved in rice drought tolerance via rhizosheath formation. Thus, we focus to explore the role of *Pseudomonas* in rice rhizosheath formation.

About 20–40% of plant photoassimilate‐derived carbon is used to produce root exudates, including sugars, amino acids, organic acids and secondary metabolites.^[^ ^76^, ^77^, ^78^ ^]^ Plant polyploidy has been shown to increase root exudation in tomato under salt stress.^[^ ^79^ ^]^ In the present study, flavonoid biosynthesis‐related pathways were significantly different between diploid and tetraploid rice under SD (Figure 3A), suggesting that flavonnoid quantity and composition in tetraploid rice contribute to rhizosheath formation. Flavonoids concentrations in root extracts and exudates were significantly elevated in tetraploid rice compared with diploid rice under SD (Figure 3B,D), which may be modulated by *OsCHS1* in polyploidy plants (Figure S15A, Supporting Information). Application of various flavonoids substantially increased root hair length and rhizosheath formation in both diploid and tetraploid rice under SD (Figure 3F; Figure S14A, Supporting Information), suggesting that flavonoid biosynthesis are likely involved in root hair growth for rhizosheath formation under SD. Recently, the incorporation of rhizosphere microbiomes is a frontier in plant breeding strategy.^[^ ^80^ ^]^ The present study suggests that flavonoids is involved in modulating *Pseudomonas* for rice drought resistance, which suggests that flavonoids‐related genes can be serve as one of the potential genes for rice breeding.

Root exudates serve as important signaling molecules that shape the microbial community composition.^[^ ^81^, ^82^, ^83^, ^84^, ^85^ ^]^ Flavonoids modulate interactions between roots and microbes.^[^ ^86^, ^87^ ^]^ In maize, secreted flavones regulate the abundance of Oxalobacteraceae in maize, thereby improving plant growth and nitrogen uptake.^[^ ^45^ ^]^ Flavonoids attract Aeromonadaceae by regulating chemotaxis genes expression, thus enhancing dehydration resistance.^[^ ^88^ ^]^ In the present study, tetraploid rice exhibited a significantly different rhizosheath community under SD compared with diploid rice (Figure 2B), likely due to elevated flavonoid biosynthesis (Figure 3B, D). The abundance of *Pseudomonas* in rhizosheath soil was significantly higher in tetraploid rice than in diploid rice under SD (Figure 2F), implicating this genus in phenotypic differences between ploidy levels. The application of various flavonoids further increased *Pseudomonas* abundance in rhizosheath soil (Figure 3E), indicating a role for flavonoids in shaping the rhizosheath microbiota and promoting specific taxa.

Genes involved in bacterial chemotaxis and flagellar assembly were identified in the PSE14 genome (Figure 4B), suggesting a molecular basis for functional interactions between tetraploid rice roots and PSE14 under SD. Flavonoids upregulated the expression of these chemotaxis and flagellar genes in PSE14 (Figure 4C), consistent with similar observations in *Aeromonas* sp. H1.^[^ ^88^ ^]^ Chemotactic behavior is primarily mediated by methyl‐accepting chemotaxis proteins (MCPs), which recognize various chemical signals, including nutrients and toxins. These MCPs interact with CheA to direct bacterial movement toward or away from specific stimuli.^[^ ^89^ ^]^ Deletion of the *cheA* gene in PSE14 significantly reduced chemotaxis toward flavonoids (Figure 4D), supporting the conclusion that *CheA* is involved in flavonoid‐mediated chemotaxis in *Pseudomonas*. Furthermore, the relative abundance of Bacillaceae in rhizosheath soil was lower in tetraploid rice compared with diploid rice under SD (Figure 2D). In contrast, inoculation with *P. indica* increased Bacillaceae abundance in both rhizosheath soil and the root endosphere of diploid rice,^[^ ^7^ ^]^ possibly due to differences in root exudates between tetraploid rice and *P. indica*‐inoculated diploid rice.

### 4.3 Auxin is Important for Polyploidy‐Promoted Rhizosheath Formation in Rice Under Soil Drying

Auxin‐induced root hair elongation plays a critical role in rhizosheath formation under SD.^[^ ^7^, ^20^, ^21^ ^]^ Auxin production represents one mechanism by which bacteria modulate root development.^[^ ^90^, ^91^ ^]^ We previously reported that growth‐promoting bacteria contribute to rhizosheath formation via auxin production.^[^ ^7^ ^]^ In this study, several IAA biosynthesis genes were identified in the genome of strain PSE14 (Table S11, Supporting Information), implying a role for PSE14 in promoting rhizosheath formation via auxin production. PSE14, a representative strain of *Pseudomonas* ASV1, demonstrated high IAA production (Figure S19C, Supporting Information), consistent with genomic predictions. Other bacteria, such as *C. culicis* and *P. polymyxa*, isolated from the barley rhizosheath, have been shown to improve yield via IAA biosynthesis.^[^ ^67^ ^]^ *Pseudomonas fluorescens* produces IAA via the indole‐3‐acetamine (IAM) pathway.^[^ ^92^ ^]^ Tryptophan‐2‐monooxygenase, encoded by the *iaaM* gene, is a key enzyme in this pathway.^[^ ^93^ ^]^ In this study, the IAA content of the Δ*iaaM* mutant was significantly lower than that of PSE14 and the complemented stain Δ*iaaM‐*c (Figure S19C, Supporting Information). Furthermore, rice inoculated with the Δ*iaaM* mutant exhibited significantly reduced rhizosheath formation under SD, due to decreased root hair (Figure 5A,B). The rhizosheath formation of Δ*iaaM‐*c was significantly increased relative to Δ*iaaM* mutant (Figure 5B), confirming that *Pseudomonas* promotes rhizosheath formation through auxin production. It is reported that *Chryseobacterium culicis* and *Paenibacillus polymyxa* co‐inoculation can significantly increase barley rhizosheath formation under field conditions,^[^ ^67^ ^]^ which suggests the connects microbial inoculation to rice rhizosheath formation in the present study (Figure S24, Supporting Information).

Plants also release other root exudates to alter rhizosphere microbiotal composition response to drought. A reduction in root exudate production may decrease the relative abundance of drought‐tolerant bacteria during periods of drought.^[^ ^94^ ^]^ Under drought conditions, abundance and activity of monoderm bacteria increases relative to diderms, thereby positively impacting plant growth.^[^ ^95^ ^]^ The drought‐induced phytohormone ABA is metabolized by rhizosphere bacteria and can alter microbial community composition to enhance drought tolerance.^[^ ^96^ ^]^ In sorghum, genes associated with salicylic acid and jasmonic acid pathways are down‐regulated during drought, contributing to the formation of a drought‐protective rhizosphere microbiome.^[^ ^97^ ^]^ Those reports offer more comprehensive insights into how the entire root microbial community enhances plant resilience under drought conditions.

In conclusion, tetraploid rice recruits *Pseudomonas* via flavonoid‐induced chemotaxis under drought conditions. *Pseudomonas* facilitates rhizosheath formation by modulating auxin‐induced root hair growth (Figure 5D). Certain polyploidy plants species, such as *Anoectochilus formosanus* Hayata, *Cannabis* and *Salvia officinalis* L, exhibit increased flavonoid production, suggesting a broader role for flavonoid‐mediated microbial recruitment.^[^ ^98^, ^99^, ^100^ ^]^ These findings provide insights into how polyploidy plants integrate beneficial soil microbes into rhizosheath formation and suggest potential strategies for breeding drought‐resistant crops.

## Conflict of Interest

The authors declare no conflict of interest.

## Author Contributions

F.Y.X. and W.F.X. planned and designed the research. F.Y.X., Y.S.W., J.Y.Y., X.Z., K.W., F.D., L.T., C.Q.B., S.C., L.Y.S., C.X.D., J.F., M.Q.X., L.L., X.Y., and J.H.G.L conducted most of the experiments. F.Y.X analyzed the data. F.Y.X., J.Y.P., J.P.L., Q.Z., Z.R.W., Y.Y.Z., H.Y.Z.Z., J.H.Z., and W.F.X. wrote the article. All authors read and approved the final manuscript.

## Supporting information



Supporting Information

Supporting Information

## Data Availability

The sequencing data were available in the NCBI BioProject database under accession number PRJNA859640 (16S rRNA gene sequencing data), PRJNA859542 (RNA sequencing data) and PRJNA914510 (genomic data).
