# Supplementary material for: Auxin‐Producing Pseudomonas Recruited by Root Flavonoids Increases Rice Rhizosheath Formation through the Bacterial Histidine Kinase Under Soil Drying
Source: Adv Sci (Weinh). 2025 Jun 19;12(34):e00607. doi: 10.1002/advs.202500607 (PMC12442626; doi:10.1002/advs.202500607)
Supplement: Supplementary file 1 — Supporting Information [file ADVS-12-e00607-s001.docx]

**Auxin-producing *Pseudomonas* recruited by root flavonoids increases rice rhizosheath formation through the bacterial histidine kinase under soil drying**

**1. Supplementary Tables 1-11**

**Table S1** Chemical composition of field soil used in the experiments.

| Soil component | Organic C (g kg^-1^) | Total N (g kg^-1^) | Total P (g kg^-1^) | Total K (g kg^-1^) | Olsen-P (mg kg^-1^) | Exchangeable K (mg kg^-1^) |
| --- | --- | --- | --- | --- | --- | --- |
|  | 30.2 | 1.19 | 0.556 | 29.3 | 39.8 | 85 |

**Table S4 The sequence number of samples.**

| **Samples** | **Sample type** | **Replicates** | **Seq_num** |
| --- | --- | --- | --- |
| WBS | Bulk soil | 1 | 55981 |
| WBS | Bulk soil | 2 | 31599 |
| WBS | Bulk soil | 3 | 70135 |
| MBS | Bulk soil | 1 | 73298 |
| MBS | Bulk soil | 2 | 64929 |
| MBS | Bulk soil | 3 | 56942 |
| WRP2 | Rhizosphere soil | 1 | 62406 |
| WRP2 | Rhizosphere soil | 2 | 64363 |
| WRP2 | Rhizosphere soil | 3 | 55495 |
| WRP4 | Rhizosphere soil | 1 | 52186 |
| WRP4 | Rhizosphere soil | 2 | 73606 |
| WRP4 | Rhizosphere soil | 3 | 55185 |
| MRH2 | Rhizosheath soil | 1 | 48859 |
| MRH2 | Rhizosheath soil | 2 | 46828 |
| MRH2 | Rhizosheath soil | 3 | 44350 |
| MRH4 | Rhizosheath soil | 1 | 59440 |
| MRH4 | Rhizosheath soil | 2 | 56758 |
| MRH4 | Rhizosheath soil | 3 | 64631 |
| WRE2 | Root endosphere | 1 | 66662 |
| WRE2 | Root endosphere | 2 | 36841 |
| WRE2 | Root endosphere | 3 | 34446 |
| WRE4 | Root endosphere | 1 | 45919 |
| WRE4 | Root endosphere | 2 | 53317 |
| WRE4 | Root endosphere | 3 | 48028 |
| MRE2 | Root endosphere | 1 | 58010 |
| MRE2 | Root endosphere | 2 | 54449 |
| MRE2 | Root endosphere | 3 | 49322 |
| MRE4 | Root endosphere | 1 | 70759 |
| MRE4 | Root endosphere | 2 | 72258 |
| MRE4 | Root endosphere | 3 | 73694 |
| WL2 | Phyllosphere | 1 | 74290 |
| WL2 | Phyllosphere | 2 | 72519 |
| WL2 | Phyllosphere | 3 | 50589 |
| WL4 | Phyllosphere | 1 | 65047 |
| WL4 | Phyllosphere | 2 | 46531 |
| WL4 | Phyllosphere | 3 | 43934 |
| ML2 | Phyllosphere | 1 | 42588 |
| ML2 | Phyllosphere | 2 | 40763 |
| ML2 | Phyllosphere | 3 | 45622 |
| ML4 | Phyllosphere | 1 | 43221 |
| ML4 | Phyllosphere | 2 | 42487 |
| ML4 | Phyllosphere | 3 | 50237 |

**Note:**

WBS, bulk soil under the well-watered (WW) conditions; MBS, bulk soil under soil drying (SD); WRE2, root endosphere of diploid rice under WW; WRE4, root endosphere of tetraploid rice under WW; MRE2, root endosphere of diploid rice under SD; MRE4, root endosphere of tetraploid rice under SD; WRP2, rhizosphere soil of diploid rice under WW; WRP4, rhizosphere soil of tetraploid rice under WW; MRH2, rhizosheath soil of diploid rice under SD; MRH4, rhizosheath soil of tetraploid rice under SD; WLP2, phyllosphere of diploid rice under WW; WLP4, phyllosphere of tetraploid rice under WW; MLP2, phyllosphere of diploid rice under SD; MLP4, phyllosphere of tetraploid rice under SD.

**Table S8** Identification of *Pseudomonas* isolates.

| Strains | Closest related species | (GenBank Acc. No.) | Similarity (%) |
| --- | --- | --- | --- |
| PSE1 | *Pseudomonas nicosulfuronedens* | MN007089 | 99.65% |
| PSE2 | *Pseudomonas nicosulfuronedens* | MN007089 | 99.72% |
| PSE3 | *Pseudomonas nicosulfuronedens* | MN007089 | 99.93% |
| PSE4 | *Pseudomonas nicosulfuronedens* | MN007089 | 99.86% |
| PSE11 | *Pseudomonas nicosulfuronedens* | MN007089 | 99.86% |
| PSE15 | *Pseudomonas nicosulfuronedens* | MN007089 | 99.65% |
| PSE16 | *Pseudomonas nicosulfuronedens* | MN007089 | 99.51% |
| PSE17 | *Pseudomonas nicosulfuronedens* | MN007089 | 99.93% |
| PSE18 | *Pseudomonas nicosulfuronedens* | MN007089 | 99.72% |
| PSE23 | *Pseudomonas nicosulfuronedens* | MN007089 | 99.79% |
| PSE29 | *Pseudomonas nicosulfuronedens* | MN007089 | 99.72% |
| PSE31 | *Pseudomonas nicosulfuronedens* | MN007089 | 99.86% |
| PSE42 | *Pseudomonas nicosulfuronedens* | MN007089 | 99.50% |
| PSE47 | *Pseudomonas nicosulfuronedens* | MN007089 | 99.58% |
| PSE5 | *Pseudomonas sp. J14* | EU099380 | 99.58% |
| PSE6 | *Pseudomonas sp. J14* | EU099380 | 99.72% |
| PSE10 | *Pseudomonas sp. J14* | EU099380 | 99.51% |
| PSE28 | *Pseudomonas sp. J14* | EU099380 | 99.30% |
| PSE30 | *Pseudomonas sp. J14* | EU099380 | 99.37% |
| PSE32 | *Pseudomonas sp. J14* | EU099380 | 99.79% |
| PSE34 | *Pseudomonas sp. J14* | EU099380 | 99.86% |
| PSE35 | *Pseudomonas sp. J14* | EU099380 | 99.65% |
| PSE44 | *Pseudomonas sp. J14* | EU099380 | 99.86% |
| PSE46 | *Pseudomonas sp. J14* | EU099380 | 99.72% |
| PSE49 | *Pseudomonas sp. J14* | EU099380 | 99.72% |
| PSE52 | *Pseudomonas sp. J14* | EU099380 | 99.78% |
| PSE53 | *Pseudomonas sp. J14* | EU099380 | 99.93% |
| PSE54 | *Pseudomonas sp. J14* | EU099380 | 99.65% |
| PSE7 | *Pseudomonas putida* | KY963635 | 99.86% |
| PSE8 | *Pseudomonas nitroreducens* | KY292456 | 99.79% |
| PSE9 | *Pseudomonas nitroreducens* | KY292456 | 99.79% |
| PSE19 | *Pseudomonas nitroreducens* | KY292456 | 99.58% |
| PSE20 | *Pseudomonas nitroreducens* | KY292456 | 99.72% |
| PSE25 | *Pseudomonas nitroreducens* | KY292456 | 99.65% |
| PSE33 | *Pseudomonas nitroreduces* | KY292456 | 99.72% |
| PSE36 | *Pseudomonas nitroreducens* | KY292456 | 99.79% |
| PSE37 | *Pseudomonas nitroreducens* | KX682023 | 99.44% |
| PSE38 | *Pseudomonas nitroreducens* | KY292456 | 99.72% |
| PSE50 | *Pseudomonas nitroreducens subsp.* | AB681730 | 99.86% |
| **PSE14** | ***Pseudomonas nitroreducens*** | **AB681730** | **99.51%** |
| PSE12 | *Pseudomonas knackmussii* | CP047698 | 99.72% |
| PSE13 | *Pseudomonas knackmussii* | CP047698 | 99.86% |
| PSE22 | *Pseudomonas monteilii* | MT823485 | 99.79% |
| PSE39 | *Pseudomonas monteilii* | MT124555 | 99.93% |
| PSE40 | *Pseudomonas monteilii* | MT124555 | 99.86% |
| PSE41 | *Pseudomonas monteilii* | GQ284481 | 99.86% |
| PSE21 | *Pseudomonas mendocina* | MW578910 | 99.93% |
| PSE24 | *Pseudomonas sp* | CP045553 | 99.79% |
| PSE45 | *Pseudomonas sp* | MH113274 | 99.79% |
| PSE48 | *Pseudomonas sp* | MW578883 | 99.72% |
| PSE51 | *Pseudomonas sp. 14181154* | CP045554 | 100% |
| PSE43 | *Pseudomonas sp. LMB-2* | KR048282 | 99.57% |
| PSE27 | *Pseudomonas sp. Fenol2C* | KU291441 | 99.72% |
| PSE26 | *Pseudomonas plecoglossicida* | DQ095898 | 99.79% |

**2. Supplementary Figures 1-16**

**Figure. S1 A,** Schematic representation of the 16S rRNA gene high-throughput sequencing. **B,** The cross section of rice rhizosheath. Bar = 50 μm. **C,** Schematic representation of the effect of PSE14 on rhizosheath formation. Diploid and tetraploid rice were inoculated with the control, 1-naphthylphthalamic acid (NPA, a polar auxin transport inhibitor) or PSE14 under SD.

**Figure. S2 A,** Plant height; B, Shoot fresh weight; C, Root fresh weight; D, Shoot dry weight; E, Root dry weight; F, Total root length; G, Root hair length; H, root hair density. Data are the means ± SE (*n* = 4). Differences were evaluated using the two-tailed Student’s *t* test (**p* < 0.05; ****p* < 0.001; n.s, not significant).

**Figure. S3 A,** The phenotype of diploid and tetraploid rice. B, Net rate of CO_2_ assimilation; C, Content of free proline; D, Content of MDA. Bar = 10 cm. Data are the means ± SE (*n* = 4). Differences were evaluated using the two-tailed Student’s *t* test (**p* < 0.05; n.s, not significant).

**Figure. S4 A-C,** Primary root elongation, root hair length and root hair density of diploid and tetraploid rice under drought conditions. Bar = 10 cm. Data are the means ± SE (*n* = 6). Differences were evaluated using the two-tailed Student’s *t* test (**p* < 0.05; ****p* < 0.001; n.s, not significant).

**Figure. S5** **A,** Intrinsic water use efficiency (iWUE) of diploid (2×) and tetraploid (4×) rice under the well-watered (WW) and soil drying (SD) conditions. Data are the means ± SE (*n* = 6). iWUE of rice leaf was calculated as the ratio of net photosynthetic rate and stomatal conductance. **B,** Correlation between rhizosheath formation and iWUE under SD. Data are the means ± SE (*n* = 6). **C,** Rhizosheath soil water content in diploid (2×) and tetraploid (4×) rice under soil drying (SD) conditions. Data are the means ± SE (*n* = 6). **D,** Root hair length of diploid (2×) and tetraploid (4×) rice under WW and SD conditions. Data are the means ± SE (*n* = 6).

Boxes span from the first to the third quartiles, center lines represent median values and whiskers show data lying within 1.5 × interquartile ranges of lower and upper quartiles. Differences were evaluated using the two-tailed Student’s *t* test (**p* < 0.05; ***p* < 0.01; ****p* < 0.001; n.s not significant).

**Figure. S6** **A,** The Olsen-P concentration in natural soil and sterilized soil. **B,** Root hair length of diploid (2×) and tetraploid (4×) rice growth in SD conditions with and without previous sterilization. **C,** Root hair length in diploid (2×) rice and tetraploid (4×) rice when inoculated with the rhizosheath microbiota of diploid rice (2×M) or the rhizosheath microbiota of tetraploid rice (4×M). All data are the means ± SE (*n* = 6). Boxes span from the first to the third quartiles, center lines represent median values and whiskers show data lying within 1.5 × interquartile ranges of lower and upper quartiles. Differences were evaluated using the two-tailed Student’s *t* test (**p* < 0.05; ***p* < 0.01; ns, not significant).

**Figure. S7** Rarefaction curves of soil bacterial community of all samples. WBS, bulk soil under the well-watered (WW) conditions; MBS, bulk soil under soil drying (SD); WRE2, root endosphere of diploid rice under WW; WRE4, root endosphere of tetraploid rice under WW; MRE2, root endosphere of diploid rice under SD; MRE4, root endosphere of tetraploid rice under SD; WRP2, rhizosphere soil of diploid rice under WW; WRP4, rhizosphere soil of tetraploid rice under WW; MRH2, rhizosheath soil of diploid rice under SD; MRH4, rhizosheath soil of tetraploid rice under SD; WLP2, phyllosphere of diploid rice under WW; WLP4, phyllosphere of tetraploid rice under WW; MLP2, phyllosphere of diploid rice under SD; MLP4, phyllosphere of tetraploid rice under SD.

**Figure. S8 A,** The alpha-diversity (based on Chao and Shannon index) of diploid (2×) and tetraploid (4×) rice under the well-watered (WW) conditions. The Wilcoxon rank-sum test was used to analyse the significance in alpha-diversity (based on Chao and Shannon index). **B,** Principal-coordinate analysis (PCoA, based on Bray-Curtis distance) showing the difference in the composition of bacterial microbiomes in bulk soil and rhizosheath soil of diploid (2×) and tetraploid (4×) rice under WW. **C-D,** The phylum-level and family-level relative abundances from rhizosphere soil of diploid (2×) and tetraploid (4×) rice under WW. RP, rhizosphere soil; Rhizosphere-2×, rhizosphere soil of diploid rice under WW; Rhizosphere-4×, rhizosphere soil of tetraploid rice under WW.

**Figure. S9 A, B,** Principal-coordinate analysis (PCoA, based on Bray-Curtis distance) of diploid (2×) and tetraploid (4×) rice in the phyllosphere (A) and root endosphere (B) under the well-watered (WW) and soil drying (SD) conditions. **C-F,** The phylum-level and family-level relative abundances of diploid (2×) and tetraploid (4×) rice in the phyllosphere (B, E) and root endosphere (C, F) under WW and SD. WL-2×, phyllosphere of diploid rice under WW; WL-4×, phyllosphere of tetraploid rice under WW; ML-2×, phyllosphere of diploid rice under SD; ML-4×, phyllosphere of tetraploid rice under SD. WRE-2×, root endosphere of diploid rice under WW; WRE-4×, root endosphere of tetraploid rice under WW; MRE-2×, root endosphere of diploid rice under SD; MRE-4×, root endosphere of tetraploid rice under SD.

**Figure. S10 A,** The relative abundance of *Pseudomonas* genus in rhizosphere soil of diploid (2×), tetraploid (4×) rice under the well-watered (WW) conditions. Data are means ± SE (*n* = 3). Differences were evaluated using the Kruskal-Wallis test (*p* < 0.05). **B,** Abundance of cultivable *Pseudomonas* in rhizosheath soil of diploid (2×) and tetraploid (4×) rice under soil drying (SD). Data are means ± SE (*n* = 6). Data were represented as median values (horizontal line), center lines represent median values and whiskers show data lying within 1.5 × interquartile ranges of lower and upper quartiles.

**Figure. S11 Volcano map showing differentially expressed genes (DEGs) between diploid (2×) and tetraploid (4×) rice under soil drying (SD) conditions.** DEGs were identified by gene-expression-level analysis with fold change values ≥ 2.

**Figure. S12 A,** Volcano plot of differentially abundant metabolites between diploid (2×) and tetraploid (4×) rice under soil drying (SD) conditions. VIP, variable importance in projection. **B,** KEGG pathways enriched in the differentially abundant metabolites between diploid (2×) and tetraploid (4×) rice under soil drying (SD) conditions.

**Figure. S13 A,** Volcano plots showing flavonoid metabolites in diploid (2×) and tetraploid (4×) rice under soil drying (SD) conditions. The abscissa is the logarithmic value of the fold change of each metabolite between diploid (2×) and tetraploid (4×) rice, and the ordinate is the VIP (variable important in projection) value. **B,** Kyoto Eencyclopedia of Genes and Genomes (KEGG) pathway classification of flavonoid metabolites in diploid (2×) and tetraploid (4×) rice under SD.

**Figure. S14 A-B,** Effects of exogenously applied flavonoids on root hair length of diploid (2×) and tetraploid (4×) rice grown in natural (A) and sterilized (B) soil under soil drying (SD). Data were represented as median values (horizontal line), center lines represent median values and whiskers show data lying within 1.5 × interquartile ranges of lower and upper quartiles. Differences were evaluated using the two-tailed Student’s *t* test (**p* < 0.05; n.s, not significant).

**Figure. S15 A,** relative expression of *OsCHS1* in the roots of diploid and tetradiploid rice under WW and SD. **B,** Relative expression of *OsCHS1* in the roots of 2× and *OsCHS1*-overexpressing lines (OE1 and OE2). Differences were evaluated using the two-tailed Student’s *t* test (****p* < 0.001). **C,** Abundance of cultivable *Pseudomonas* in the rhizosheath soil of diploid (2×), tetraploid (4×) and *OsCHS1*-overexpressing lines OE1 rice under soil drying (SD). All data are means ± SE (*n* = 3). Differences were evaluated using the two-tailed Student’s *t* test (**p* < 0.05).

**Figure. S16 A,** The root flavonoid concentration after 21, 28 and 35 days of SD treatments; **B,** The abundance of culturable *Pseudomonas* after 21, 28 and 35 days of SD treatments; **C,** Correlation between root flavonoid concentration and abundance of culturable *Pseudomonas* under SD conditions. All data are means ± SD (*n* = 6). Differences were evaluated using the two-tailed Student’s *t* test (**p* < 0.05).

**Figure. S17** **A,** Cladogram showing phylogenetic relationships between 54 rhizosheath soil *Pseudomonas* spp. as well as ASV1. A total of 54 *Pseudomonas* strains were randomly isolated and identified from rhizosheath soil in tetraploid rice under soil drying (SD). Leaf labels show representative sequence IDs. **B,** Overview of the strain PSE14 genome. The circles represent (from outside to inside): circle 1, genome size; circle 2 and 3, genes existing in the PSE14 genome with different Cluster of Orthologous Groups of proteins (COG) function; circle 4, ncRNA; circle 5, GC-content; circle 6, GC-skew.

**Figure. S18** **Flagellar assembly pathway in the strain PSE14 genome. Different colors represent gene number.** MotA, MotB: Flagellar motor protein. FliM, FliG and FliN: Flagellar motor switch protein; FliD: Flagellar hook-associated proteins2; FliC: Flagellin protein; FlgL and FlgK: Flagellar hook-associated protein; FlgE: Flagellar hook protein; FlgD: Flagellar basal-body rod modification protein; FliK: Flagellar hook-length control protein; MotY: Sodium-type flagellar protein; FlgF and FlgG: Flagellar basal-body rod protein; FlgH: Flagellar L-ring protein precursor; FlgI: Flagellar P-ring protein precursor; FliE: Flagellar hook-basal body complex protein; FlgB and FlgC: Flagellar basal-body rod protein; FlhA and FlhB: Flagellar biosynthesis protein; FliF: Flagellar M-ring protein; FliH: Flagellar assembly protein; FliI: Flagellum-specific ATP synthase; FliO: Flagellar protein; FliQ, FliP and FliR: Flagellar biosynthetic protein; FlgN: Flagella synthesis protein; FlgA: Flagella basal body P-ring formation protein; FliJ and FliS: Flagellar protein; FlgM: Negative regulator of flagellin synthesis; FliA: RNA polymerase sigma factor for flagellar operon. Unigenes of these proteins are listed in Tables S8.

**Figure. S19 A-B,** Identification of chemotaxis mutant △*cheA* (A) and IAA biosynthesis mutant △*iaaM* (B) in the genetic background of *P.* *nitroreducens*. **C,** IAA content of the PSE14, △*iaaM* and complemented strain (△*iaaM-c*). Data are means ± SD (*n* = 3). Differences were evaluated using the two-tailed Student’s *t* test (****p* < 0.001, ns not significant).

**Figure. S20** Relative expression of flagellar genes in PSE14 and IAA biosynthesis mutant △*iaaM*. Data are the means ± SE (*n* = 3). Differences were evaluated using the two-tailed Student’s *t* test. n.s, not significant.

**Figure. S21** Root IAA concentration of diploid and tetraploid rice. Data are the means ± SD (*n* = 3). Differences were evaluated using the two-tailed Student’s *t* test (ns not significant).

**Figure. S22** Root hair length, rhizosheath formation and water-use efficiency (WUE) of diploid and tetraploid rice with the inoculation of PSE14, NPA (1-naphthylphthalamic acid, a polar auxin transport inhibitor), their combination (PSE+NPA), or (Control). Data are the means ± SE (*n* = 6). Data were represented as median values (horizontal line), center lines represent median values and whiskers show data lying within 1.5 × interquartile ranges of lower and upper quartiles. Differences were evaluated using the two-tailed Student’s *t* test (**p* < 0.05; ***p* < 0.01).

**Figure. S23** Abundance of cultivable *Pseudomonas* with inoculation of PSE14, △*iaaM* and △*iaaM-c* strain. Data and error bars are the means ± SD (*n* = 6). Differences were evaluated using the two-tailed Student’s *t* test (n.s: not significant).

**Figure. S24** Phenotype (A), shoot dry weight (B), tiller number (C) and grain yield (D) of diploid rice with the inoculation of PSE14 or Control under SD. Data are the means ± SD (*n* = 3). Differences were evaluated using the two-tailed Student’s *t* test (**p* < 0.05; ***p* < 0.01).
